# Assessment of the roles of Spt5-nucleic acid contacts in promoter proximal pausing of RNA polymerase II

**DOI:** 10.1016/j.jbc.2023.105106

**Published:** 2023-07-28

**Authors:** Roberta Dollinger, Eilene B. Deng, Josie Schultz, Sharon Wu, Haley R. Deorio, David S. Gilmour

**Affiliations:** 1 Center for Eukaryotic Gene Regulation, Department of Biochemistry and Molecular Biology, Pennsylvania State University, University Park, Pennsylvania, USA; 2 Department of Biomolecular Chemistry, University of Wisconsin-Madison, Madison, Wisconsin, USA

**Keywords:** RNA polymerase II, promoter proximal pausing, DSIF, Spt5, NGN domain, NELF, mRNA, RNA-protein interaction, DNA–protein interaction, transcription

## Abstract

Promoter proximal pausing of RNA polymerase II (Pol II) is a critical transcriptional regulatory mechanism in metazoans that requires the transcription factor DRB sensitivity-inducing factor (DSIF) and the inhibitory negative elongation factor (NELF). DSIF, composed of Spt4 and Spt5, establishes the pause by recruiting NELF to the elongation complex. However, the role of DSIF in pausing beyond NELF recruitment remains unclear. We used a highly purified *in vitro* system and *Drosophila* nuclear extract to investigate the role of DSIF in promoter proximal pausing. We identified two domains of Spt5, the KOW4 and NGN domains, that facilitate Pol II pausing. The KOW4 domain promotes pausing through its interaction with the nascent RNA while the NGN domain does so through a short helical motif that is in close proximity to the non-transcribed DNA template strand. Removal of this sequence in *Drosophila* has a male-specific dominant negative effect. The alpha-helical motif is also needed to support fly viability. We also show that the interaction between the Spt5 KOW1 domain and the upstream DNA helix is required for DSIF association with the Pol II elongation complex. Disruption of the KOW1–DNA interaction is dominant lethal *in vivo*. Finally, we show that the KOW2-3 domain of Spt5 mediates the recruitment of NELF to the elongation complex. In summary, our results reveal additional roles for DSIF in transcription regulation and identify specific domains important for facilitating Pol II pausing.

Eukaryotic transcription is a highly regulated process that depends on the precise spatiotemporal coordination of multiple interacting factors at each stage of the transcription cycle. Initiation, elongation, and termination have long been regarded as the primary canonical steps of this cycle. However, promoter proximal pausing of RNA polymerase II (Pol II) is now recognized as an additional critical post-initiation step in metazoan transcription. Promoter proximal pausing is characterized by an accumulation of Pol II ∼30 to 60 nucleotides downstream of the transcription start site (1). This phenomenon was first observed as a concentration of transcriptionally engaged Pol II at the 5′ end of the beta-globin gene in nuclei from mature hen erythrocytes that were expected to be transcriptionally silent (2). Several subsequent studies led to the observation of similar phenomena on mammalian *c-myc* (3, 4, 5) and HIV-1 (6), as well as at non-induced *Drosophila* heat shock genes (7, 8). The work by Gilmour and Lis on the *Drosophila hsp70* gene established that a single Pol II molecule associates with the non-induced *hsp70* gene in the region between −12 and +65 (7) and subsequent experiments by Rougvie and Lis demonstrated that this Pol II is transcriptionally engaged (8). Since then, genomic methods have provided overwhelming evidence that promoter proximal pausing is a ubiquitous step in the transcription cycle for most *Drosophila* and mammalian protein-coding genes (9, 10, 11, 12). Pausing is associated with several critical regulatory functions, including developmental control and the maintenance of a nucleosome-free, permissive chromatin architecture around promoters (11, 13, 14, 15).

Promoter proximal pausing requires DRB sensitivity-inducing factor (DSIF) and negative elongation factor (NELF), two factors that function cooperatively to establish the pause (16, 17, 18, 19). DSIF is a widely conserved eukaryotic transcription factor that associates with the elongation complex after the transcription of at least 18 nucleotides (19). The role of DSIF in pausing was first identified as an activity that rendered Pol II transcription sensitive to inhibition by the nucleoside analog 5,6-dichloro-1-β-D-ribofuranosylbenzimidazole (DRB) (16). NELF was identified as an inhibitory factor that, together with DSIF, works to repress metazoan Pol II transcription (17, 20). Release of the pause and the transition to productive elongation is thought to be mediated by the cyclin-dependent kinase positive transcription elongation factor b (P-TEFb), which phosphorylates Pol II, DSIF, and NELF, resulting in the ejection of NELF from the elongation complex and the transformation of DSIF from a negative to a positive elongation factor (18, 21, 22, 23).

A structure of the human paused elongation complex from Cramer and colleagues containing Pol II, DSIF, and NELF sheds light on the possible mechanisms by which NELF induces the pause (24). In this model, NELF stabilizes the formation of a half-translocated RNA–DNA duplex in the active site, preventing an incoming nucleotide from base pairing with the template. Furthermore, the interaction between NELF-C and the open Pol II trigger loop may interfere with trigger loop folding, which is needed to close off the active site and facilitate nucleotide addition (24, 25, 26). However, the role of DSIF in promoter proximal pausing has been less clear. DSIF is the lynchpin of the paused elongation complex because it is required to recruit NELF (19), but how the interactions between DSIF and the Pol II elongation complex contribute to pausing remains ambiguous. Several *in vitro* studies using highly purified systems indicate that on its own, DSIF either has no effect or a slight stimulatory effect on transcription (17, 19, 24, 27). Hence, whether DSIF serves solely as an adapter that recruits regulators of elongation or itself contributes to pausing is an open question.

Of particular interest are the interactions between the Spt5 subunit and the nucleic acid scaffold. Spt5 has several domains, including unstructured N- and C-terminal regions, a NusG N-terminal (NGN) domain, and several Kyprides, Ouzounis, Woese (KOW) domains (Fig. 1*A*). Structures of the human elongation complex revealed that the NGN and KOW1 domains form part of the upstream DNA exit tunnel and that the KOW4 and KOW5 domains form a clamp around the nascent transcript (Fig. 1, *A* and *B*) (24, 28, 29). Comparison of the Spt5 conformations between cryo-EM structures of the paused and active elongation complexes highlights a repositioning of the KOW1 and KOW4 domains upon pause release, resulting in an opening of the nucleic acid clamps (Fig. 1*B*) (24, 29). Translocation of Pol II requires the movement of the nucleic acids through their respective exit channels. For Pol II to move along the DNA, the upstream DNA must be able to exit though the upstream DNA exit channel, the mouth of which is framed by the Spt5 DNA clamp, and the nascent transcript must exit through the Spt5 RNA clamp (Fig. 1*A*) (24, 29).

**Figure 1:**
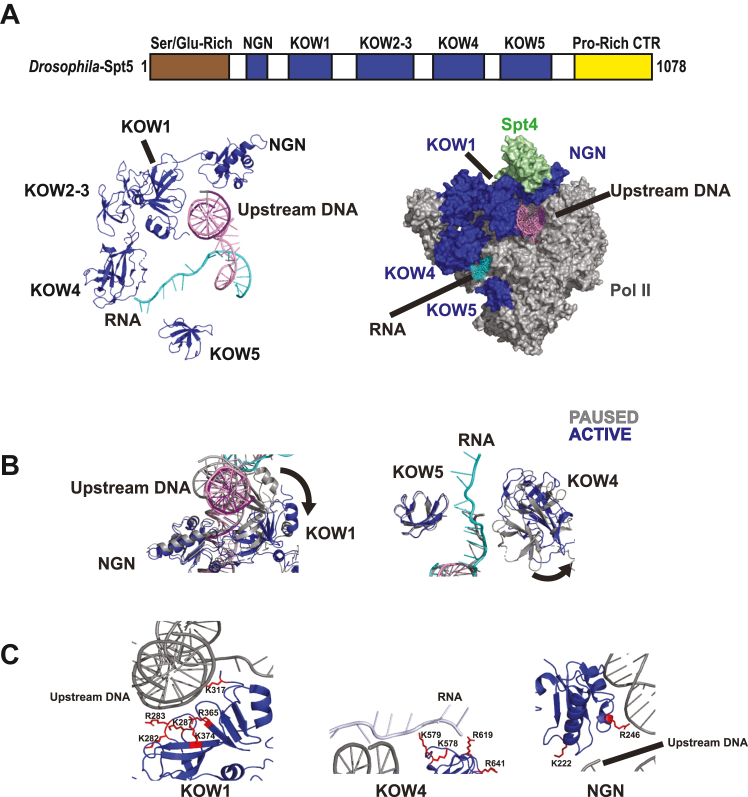
**Architecture of Spt5 Cartoon (*Drosophila*) and structural (human) illustrations of Spt5 in the context of the nucleic acids and the Pol II elongation complex**. *A*, the Spt5 subunit consists of an N-terminal Ser/Glu-rich domain, an NGN domain, several KOW domains, and a Pro-rich C-terminal region (*top*). The NGN and KOW1 domains form a portion of the upstream DNA exit tunnel while the KOW4 and KOW5 domains form a clamp around the nascent RNA (*bottom*). *B*, upon the transition of the elongation complex from the paused to the active state, both nucleic acid clamps undergo a conformational change. The KOW1 and KOW4 domains rotate to result in the opening of the DNA and RNA clamps respectively. *C*, the interactions between the NGN, KOW1, and KOW4 domains and the nucleic acids are mediated by positively charged residues. PDB ID: 6GML, 6GMH.

We hypothesized that Spt5–nucleic acid interactions facilitate promoter proximal pausing by restricting the movement of the upstream DNA and nascent RNA through their exit channels (Fig. 1, *A* and *C*). To test our hypothesis, we generated DSIF mutants in which the charges of basic nucleic acid-interacting residues of Spt5 were reversed. To identify the pausing functions of the Spt5-nucleic acid contacts, we used a highly purified *in vitro* system to screen these mutants for Pol II binding and NELF recruitment. We then tested each mutant’s ability to rescue promoter proximal pausing in *Drosophila* nuclear extract depleted of wild-type DSIF. We found that the contacts between the KOW1 domain and the upstream DNA mediate the association of DSIF with the elongation complex; since DSIF binding to the elongation complex is a prerequisite for NELF recruitment, the KOW1-DNA interaction thus governs promoter proximal pausing indirectly. Furthermore, the expression of the Spt5 KOW1 mutant is lethal in *Drosophila.* In contrast, the interactions between the KOW4 domain and the nascent transcript directly facilitate promoter proximal pausing. We also identified a short helical motif in the NGN domain that is critical to facilitating the pause. This sequence is highly conserved in eukaryotes that encode NELF but notably absent in eukaryotes that lack promoter proximal pausing and NELF. In flies, the replacement of this helical motif with homologous sequences from *Saccharomyces cerevisiae* and *Caenorhabditis elegans* results in a male-specific dominant negative effect. Spt5 NGN mutants also fail to support *Drosophila* viability when wild-type Spt5 has been depleted with RNAi. Taken together, our results provide a functional assessment of the various domains of Spt5.

## Results

### Purification of Pol II, NELF, and DSIF

We first generated high-purity preparations of Pol II and each of the factors involved in promoter proximal pausing, NELF and DSIF. Pol II was purified from *Drosophila* embryos, NELF from a baculovirus expression system, and DSIF from a previously established *Escherichia coli* expression system (30).

**Figure 2:**
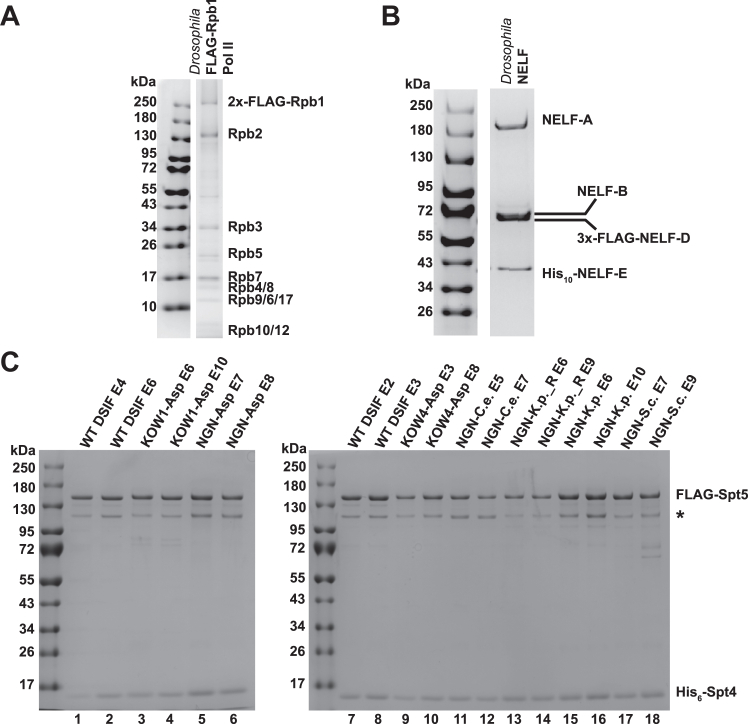
**Purified Pol II, NELF, and DSIF Coomassie-stained 3% to 14% Tris-Acetate gels**. *A*, Pol II was purified from *Drosophila* embryo nuclear extract. The fly line used contains a FLAG tag on the C-terminus of the Rpb1 subunit, allowing for affinity purification over an anti-FLAG column. *B*, NELF was purified from Sf9 cells infected with a baculovirus containing the coding sequences for the four NELF subunits, with a 3x-FLAG tag on the C-terminus of NELF-D and a His_10_ tag on the N-terminus of NELF-E. *C*, WT DSIF and DSIF mutants were purified from *E. coli* using an expression plasmid containing the coding sequences for *Drosophila* Spt5 and Spt4 with a FLAG tag on the Spt5 C-terminus and a His_6_ tag on the C-terminus of Spt4. This allowed tandem affinity purification over a metal affinity column followed by an anti-FLAG column. ∼200 ng of Spt5 were loaded in each lane. A ∼100-kDa Spt5 degradation product lacking the acidic N-terminal region appears in each preparation (*asterisk*) (30).

We purified Pol II from a fly line with an endogenous FLAG tag on the C-terminal domain of the Rpb1 subunit (FLAG-Rpb1) (31). Nuclear extract was generated from *Drosophila* embryos (30, 32, 33), and Pol II was enriched by passing the extract over a POROS Heparin column. Pol II was then purified using an anti-FLAG column. As shown in Figure 2*A*, this resulted in a highly purified preparation of Pol II with all subunits visible on a Coomassie-stained Tris-Acetate gel (Fig. 2*A*). Using the FLAG-Rpb1 fly line as a source of Pol II also ensured that the final purified product contains an Rpb1 subunit with an intact carboxyl-terminal domain (CTD). A portion of the Rpb1 in the nuclear extracts is missing the CTD due to proteolysis (Fig. S1*A*) but this is removed by the final purification step.

We purified NELF from baculovirus-infected Sf9 cells using a baculovirus encoding all four subunits (NELF-A, NELF-B, NELF-D, and NELF-E) of *Drosophila* NELF. To purify the complex, we elected to insert a His_10_ tag on the N-terminus of NELF-E and a 3x-FLAG tag on the C-terminus of NELF-D. This allowed for rapid tandem purification using a Nickel-NTA column followed by an anti-FLAG column. The result was a highly purified, concentrated preparation with all four NELF subunits (Fig. 2*B*).

DSIF and DSIF mutants were expressed in *E. coli* using a pST44-Spt5-Spt4 polycistronic expression vector containing the coding sequences for *Drosophila* Spt5 and Spt4 with a 3x-FLAG tag on the C-terminus of Spt5 and a His_6_ tag on the C-terminus of Spt4 (30, 34). This allowed for tandem purification with a TALON column followed by an anti-FLAG column as previously described (30). We successfully obtained highly purified preparations of WT DSIF and a total of nine DSIF mutants. Figure 2*C* shows Coomassie-stained gels with multiple purified fractions of WT and mutant DSIF. An Spt5 degradation product lacking the Ser- and Glu-rich N-terminal domain appears in each preparation (Fig. 2*C*, asterisk) (30). DSIF preparations were quantified by running WT DSIF and DSIF mutants on 3 to 14% Tris-Acetate gels next to BSA standards and comparing full-length Spt5 band intensities to a BSA standard curve.

### Nucleic acid contacts made by the KOW1 domain of Spt5 mediate Pol II-DSIF binding

The KOW1 domain of Spt5 forms one-half of a nucleic acid clamp that brackets the upstream DNA. Analysis of structural data of the paused and active elongation complexes indicates that the Spt5 DNA clamp opens as a result of the repositioning of the KOW1 domain when Pol II transitions from a paused state to an active state (Fig. 1*B*) (24, 29). We hypothesized that the closed DNA clamp facilitates promoter proximal pausing by restricting the movement of the upstream DNA through the exit channel, thus preventing the Pol II from translocating. To test this, we used the existing structural data for the human paused elongation complex to identify basic residues that facilitate Spt5–nucleic acid interactions (24) and determined which of these residues are conserved in *Drosophila.* The KOW1 domain has six conserved positively charged residues close to the upstream DNA: K317 (dR354), R365 (dR405), K374 (dK311), K287 (dK324), K282 (dK319), and R283 (dR320) (Fig. 1*C*). To test the hypothesis that interactions between basic residues and the upstream DNA facilitate pausing, we disrupted the interactions by mutating all six residues in *Drosophila* Spt5 to aspartic acid. We will subsequently refer to this mutant as the KOW1-Asp mutant.

**Figure 3:**
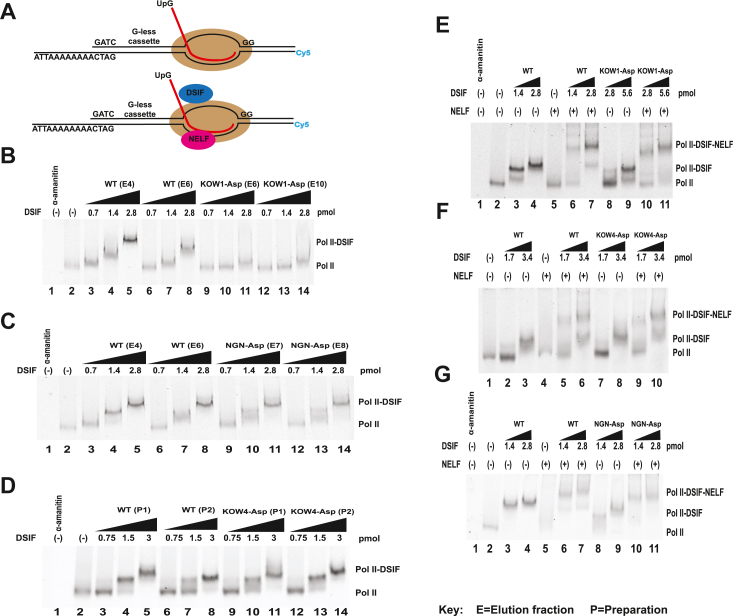
**Binding activity of Spt5 charge reversal mutants.***A*, stalled elongation complexes were generated using FLAG-Rpb1 Pol II and a tailed, fluorescently labeled DNA template containing a G-less cassette. DSIF and/or NELF were then added, and complexes were evaluated on a 4% native polyacrylamide gel. *B*, KOW1-Asp mutant binding to Pol II. Two separate mutant fractions (E6 and E10) were compared to two separate WT DSIF (E4 and E6) fractions. *C*, NGN-Asp Pol II binding. Two separate mutant fractions (E7 and E8) were compared to two separate WT DSIF fractions (E4 and E6). *D*, KOW4-Asp mutant binding to Pol II. Two separate preparations of KOW4-Asp (P1 and P2) were compared to two separate preparations of WT DSIF (P1 and P2). *E*, Pol II and NELF binding of KOW1-Asp mutant. *F*, Pol II and NELF binding of KOW4-Asp. *G*, Pol II and NELF binding of NGN-Asp mutant. Additional replicates can be found in Figs. S2 and S3.

We first tested the ability of the KOW1-Asp mutant to bind Pol II and recruit NELF to the elongation complex. Promoter proximal pausing depends on the capacity of DSIF to associate with the elongation complex and recruit NELF (30, 33). To determine which of these functions is affected in the KOW1-Asp mutant, we performed electrophoretic mobility shift assays (EMSAs). We used purified *Drosophila* Pol II (Fig. 2*A*) and a Cy5-labeled tailed template with a G-less cassette to generate elongation complexes with 26-nucleotide-long transcripts. Purified wild-type or mutant DSIF (Fig. 2*C*) and NELF (Fig. 2*B*) were then added and the complexes were analyzed on native polyacrylamide gels (Fig. 3*A*) (19, 30).

As shown in previous studies (19, 30), the addition of WT DSIF produced a clear upward mobility shift of the elongation complex, commensurate with the amount of DSIF added. Figure 3 shows representative EMSA experiments. The addition of 0.7 pmol did not shift the complex. Addition of 1.4 pmol of WT DSIF resulted in an incomplete shift of the elongation complex and the addition of 2.8 pmol produced a complete shift (Fig. 3*B*, lanes 4–5, 7–8). However, the KOW1-Asp mutant exhibited a marked decrease in its ability to bind Pol II. The addition of 1.4 pmol of the KOW1-Asp mutant failed to shift the elongation complex and 2.8 pmol of the mutant produced incomplete shifting (Fig. 3*B* lanes 10–11, 13–14). The addition of 5.6 pmol of KOW1-Asp mutant shifted most, but not all, of the elongation complex (Fig. 3*E*, lane 9). To control for variation in the quantification of Spt5, Pol II binding EMSAs were performed with at least two different fractions of WT DSIF and two different fractions of the mutant. The results showing decreased Pol II binding by KOW1-Asp were reproducible across multiple fractions from multiple protein preparations (Figs. 3*B* and S2*A*). These results indicate that the electrostatic interactions between the KOW1 domain and the upstream DNA facilitate DSIF binding to the Pol II elongation complex.

We also used this EMSA to test the ability of KOW1-Asp to recruit NELF to the elongation complex. The addition of NELF alone did not shift the Pol II elongation complex (Fig. 3*E*, lane 5), consistent with a previous study (19). The addition of WT DSIF and NELF together supershifted the complex, with 2.8 pmol of WT DSIF (full elongation complex shift) producing a sharper supershift band than 1.4 pmol (Fig. 3*E*, cf lanes 6 and 7). Despite having a lower affinity for the Pol II elongation complex, KOW1-Asp DSIF was able to produce a NELF supershift if the mutant DSIF was added in sufficient quantities to detect its association with the elongation complex (Fig. 3*E*, lanes 10 and 11, Fig. S3*A*, lanes 10 and 11), and the percentage of NELF-containing complexes formed was comparable to that of WT DSIF (Fig. S4*A*). These results demonstrate that KOW1-Asp has a decreased affinity for the Pol II elongation complex relative to WT DSIF but is still capable of recruiting NELF if present in higher quantities.

**Figure 4:**
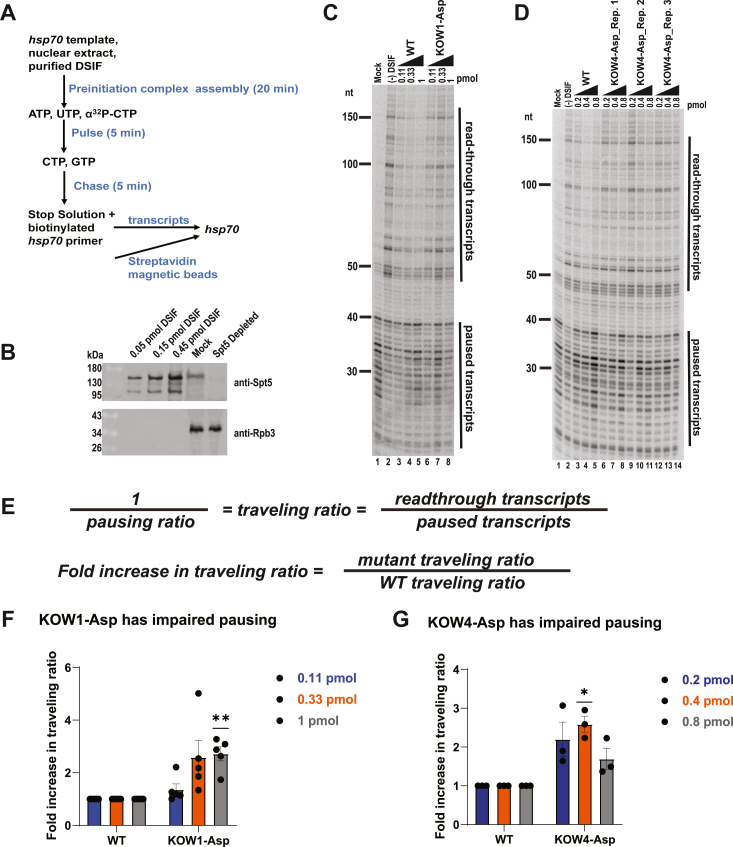
**DSIF charge reversal mutants have impaired pausing in nuclear extract.***A*, purified wild-type or mutant DSIF was added to *Drosophila* nuclear extract immunodepleted of endogenous DSIF. Transcription reactions were carried out using an *hsp70* template. Reactions with mock depleted extract were set up as controls. RNA was isolated using a biotinylated primer and analyzed on a 10% Urea-PAGE gel. *B*, Western blot against Spt5 and Pol II subunit Rpb3 in mock depleted and DSIF depleted nuclear extracts; each lane contains 7.5 uL of extract. 0.05, 0.15, and 0.45 pmol of recombinant DSIF were loaded for comparison. Note that the transcription reactions use 16 ul of extract. *C*, pausing activity of KOW1-Asp mutant. *D*, pausing activity of KOW4-Asp mutant. *E*, pausing assay gels were quantified by calculating traveling ratios for each reaction. The traveling ratio is equal to the signal from readthrough transcripts divided by the signal from paused transcripts. The fold increase in traveling ratio relative to WT DSIF was then calculated for each mutant. *F*, average fold increase in the traveling ratio for KOW1-Asp (*panel C*). Additional replicates can be found in Fig. S5 (n = 5). Two stars indicate *p* < 0.01. Error bars indicate the standard error of the mean. *G*, average fold increase in traveling ratio for KOW4-Asp (*panel D*). One star indicates *p* < 0.05 (n = 3). Error bars indicate the standard error of the mean.

To measure the pausing activity of the KOW1-Asp mutant, we carried out *in vitro* transcription reactions in nuclear extract immunodepleted of WT DSIF (Fig. 4*A*). Nuclear extracts from *Drosophila* embryos were mock depleted or DSIF-depleted with pre-immune serum or Spt5 antibody, respectively. The depletion was verified by Western Blot (Fig. 4*B*). We then performed transcription reactions using a *Drosophila hsp70* promoter template and tested the ability of WT and mutant DSIF to restore pausing in the depleted extract (Fig. 4*A*) (30, 33). The mock depleted extract primarily produced paused transcripts of less than 40 nucleotides (nt) in length (Fig. 4*C*, lane 1). Depleting DSIF resulted in a significant increase in the proportion of read-through transcripts (Fig. 4*C*, lane 2), indicating impaired pausing. To quantify the difference in pausing ability between the KOW1-Asp mutant and WT DSIF, we divided the signal from readthrough transcripts by the signal from paused transcripts to generate a traveling ratio for each transcription reaction (11, 12, 35). This allowed us to calculate the fold increase in traveling ratio for each mutant reaction relative to WT DSIF (Fig. 4*E*). Adding increasing amounts of purified WT DSIF leads to a decrease in traveling ratio, indicating increased pausing (Fig. 4*C*, lanes 3–5, Figure 4*D*, lanes 3–5, Figs. S5 and S6, WT lanes). However, KOW1-Asp exhibited a marked pausing impairment (Fig. 4*C*, cf lanes 3–5 to 6–8, Fig. S5, *A*–*E*). At low quantities of added DSIF (*i.e.* 0.111 pmol), we observed only a slight increase in KOW1-Asp traveling ratio relative to WT DSIF (Fig. 4*F*). This can be explained by the incomplete restoration of pausing in immunodepleted nuclear extract by low quantities of WT DSIF (Fig. S5, *B*–*E*, lane 3). However, at higher quantities (0.333 pmol or 1 pmol DSIF) we found that KOW1-Asp exhibited an average of ∼2.5-fold increase in traveling ratio relative to WT DSIF (n = 5, *p* < 0.01 for 1 pmol) (Fig. 4*F*). We propose the decreased pausing activity of KOW1-Asp is a result of its decreased affinity for the Pol II elongation complex, which would result in a decrease in the proportion of complexes able to bind NELF. An additional possibility is that under the conditions of the nuclear extract, the KOW1-Asp mutant is not able to form a complex with Pol II even if the mutant is added in saturating amounts. This would preclude the recruitment of NELF and result in impaired Pol II pausing. Moreover, although the EMSA indicates that KOW1-Asp can mediate the binding of NELF when a sufficient amount of KOW1-Asp is present (Fig. 3*E*), it is unlikely that there is a match between the amount of DSIF required for NELF recruitment in a purified system and for pausing in nuclear extract given the significant differences in the composition of the reactions.

### Disrupting KOW4-RNA contacts impairs promoter proximal pausing

The KOW4 domain of Spt5 forms one-half of the RNA clamp. Superimposition of the structures of the human paused and active elongation complexes reveals that the KOW4 domain changes conformation when Pol II transitions from the paused state to the active state, resulting in an opening of the Spt5 RNA clamp (Fig. 1*B*) (24, 29). Since translocation of Pol II requires the nascent transcript to move through the Spt5 RNA clamp, we hypothesized that the interactions between the KOW4 domain and the transcript in the closed conformation contribute to promoter proximal pausing.

To test this hypothesis, we used the structure of the human paused elongation complex to identify basic RNA-interacting residues in the KOW4 domain (24) and determined which of these are conserved in *Drosophila.* To disrupt the KOW4-RNA interaction, we replaced four residues, K578 (d619), K579 (dK620), R619 (dR660), and R641 (dR682) (Fig. 1*C*), with aspartic acid, generating a KOW4-Asp mutant.

We tested KOW4-Asp Pol II binding and NELF recruitment with EMSA and found that the mutant was able to bind Pol II (Fig. 3*D*, lanes 9–14 and 3F, lanes 7 and 8) and recruit NELF (Fig. 3*F* lanes 9 and 10). The KOW4-Asp affinity for the Pol II elongation complex was comparable to that of WT DSIF (Figs. 3*D* and S2*B*, cf lanes 3–5 and 6–8 to lanes 9–11 and 12–14) and its NELF recruitment activity was comparable to that of WT DSIF (Fig. 3*F*, cf lanes 6 and 10, Fig. S4*E*). Evaluation of NELF recruitment was focused on lanes in which a saturating quantity of DSIF was added (Figs. 3*F* and S3*E*, lanes 6 and 10).

We then tested the ability of the KOW4-Asp mutant to restore promoter proximal pausing in DSIF-depleted *Drosophila* nuclear extract. We tested the addition of the KOW4-Asp to DSIF-depleted nuclear extract in twofold increments (0.2 pmol, 0.4 pmol, 0.8 pmol) and observed as much as a 2.5-fold increase in KOW4-Asp traveling ratio relative to WT DSIF (n = 3 *p* < 0.05 for 0.4 pmol), indicating a reduction in pausing activity (Fig. 4, *D* and *G*). Since KOW4-Asp is able to bind Pol II and recruit NELF to a degree comparable to WT DSIF, the observed pausing impairment indicates that the interactions between the basic residues of the KOW4 domain and the nascent transcript contribute to promoter proximal pausing in a mechanism that is distinct from DSIF’s role in NELF recruitment.

### A short helical motif in the NGN domain is required for promoter proximal pausing

The NGN domain forms the second half of the upstream DNA clamp. We hypothesized that when the Spt5 DNA clamp is in the closed conformation (Fig. 1*B*), NGN-DNA interactions stabilize Pol II in the paused state. Based on the available structure (24), we identified two conserved basic residues in the NGN domain that are in close proximity to the DNA scaffold. One of these residues, K222 (dK259) contacts the upstream DNA helix while the other, R246 (dR283) is oriented toward the non-transcribed template strand (Fig. 1*C*). To disrupt the interactions between these residues and the DNA, we generated a mutant where both residues were replaced with aspartic acid, termed NGN-Asp. We first used EMSA to test the ability of NGN-Asp to bind to Pol II. Unlike the KOW1-Asp mutant, the NGN-Asp mutant was able to bind the Pol II elongation complex as well as WT DSIF, and this was consistent across multiple fractions of WT DSIF and NGN-Asp (Figs. 3*C* and S2*C*, cf lanes 9–14 to lanes 3–8). When we added NELF to the Pol II-NGN-Asp complexes, we observed a clear supershifting of the elongation complex, comparable with what was observed with WT DSIF (Figs. 3*G* and S3*B*, cf lanes 10 and 11 to lanes 6 and 7, Fig. S4*B*).

We then tested the ability of NGN-Asp to restore promoter proximal pausing in WT DSIF-depleted nuclear extract. We found that the NGN-Asp mutant had slightly impaired pausing activity relative to WT DSIF, with a ∼1.5 to 2-fold increase in traveling ratio (Fig. S6, *A*, *D*, *F*, *H* and *J*). Although this increase was not statistically significant, we observed a clear trend: the majority of our NGN-Asp replicates had an increased traveling ratio relative to the WT DSIF controls (Fig. S6*J*).

**Figure 5:**
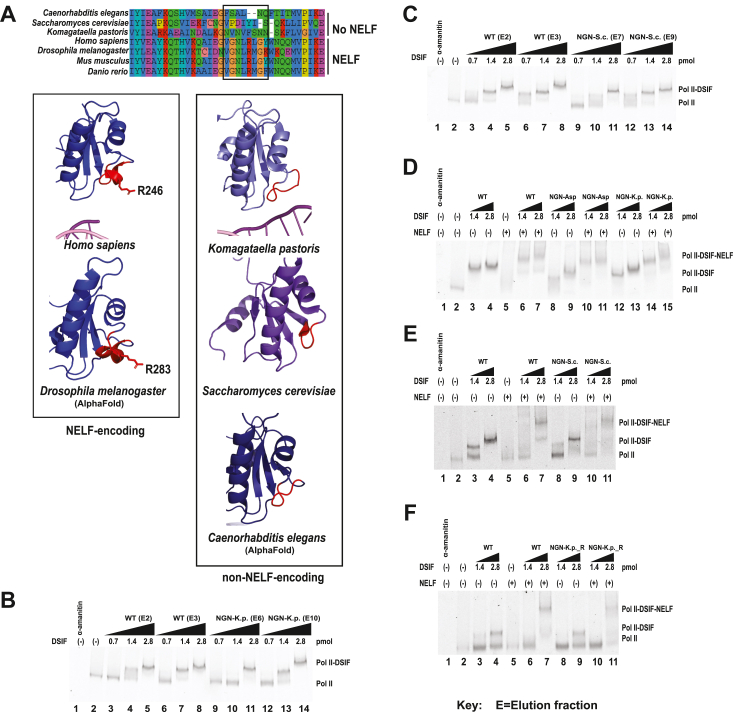
**NGN helix replacement mutants bind Pol II and recruit NELF.** Stalled elongation complexes were generated as in Figure 3. Additional replicates can be found in Figs. S2 and S3. *A*, an alpha-helix sequence in the NGN domain is conserved in NELF-expressing species but not in species without NELF. This sequence contains a conserved arginine that is mutated to aspartic acid in the NGN-Asp mutant. In species such as *K. pastoris* and *C. elegans* (predicted), this region is an unstructured loop. PDB ID: 6GML, 5XOG, 2EXU. *Drosophila* and *C. elegans* structures were modeled using Alphafold (65, 66). *B* and *C*, Pol II binding of NGN-*K. pastoris* (K.p.) and NGN-*S. cerevisiae* (NGN-S.c.) mutants. Two different mutant fractions were compared with two different WT DSIF fractions. *D*, Pol II and NELF binding of NGN-Asp and NGN-K.p. mutants. *E*, Pol II and NELF binding of NGN-S.c. *F*, Pol II and NELF binding of NGN-*K. pastoris* mutant with the conserved R246 (d283) reintroduced (NGN-K.p._R).

To follow up on this result, we first performed a sequence alignment to determine the degree of conservation of the mutated residues. K222 (dK259) is largely conserved regardless of whether a species expresses NELF and is flanked by sequences that are mostly conserved in both NELF and non-NELF species. However, R246 (dR283) is only conserved in eukaryotes with NELF and promoter proximal pausing. A closer examination reveals that this basic residue is located in the middle of a sequence conserved only in NELF-encoding species: GNL**R**(L/M)G(Y/K/F)W (Fig. 5*A*). To determine the structural significance of this difference in sequence composition between NELF-encoding and non-NELF-encoding species, we compared the NGN domain of human and *Drosophila* Spt5 to the NGN domains of yeast and worms. In the human structure and the modeled *Drosophila* structure, the conserved GNL**R**(L/M)G(Y/K/F)W sequence is structured as a short alpha helix that orients the arginine toward the non-transcribed template DNA strand. In the *Komagataella pastoris* yeast (36) structure and the modeled *C. elegans* structure, the homologous region is an unstructured loop. In the structure of the *S. cerevisiae* Spt5 NGN domain, this region is a loop that forms a turn into a beta-sheet (Fig. 5*A*) (37). Thus, we hypothesized that the conserved helical motif facilitates promoter proximal pausing.

To test our hypothesis, we generated *Drosophila* DSIF mutants in which the conserved helical motif was replaced the corresponding sequences from *K. pastoris*, *C. elegans*, and *S. cerevisiae*, termed NGN-K.p., NGN-C.e., and NGN-S.c., respectively (Fig. 2*C*). To ensure that any change in the pausing activity of these mutants relative to WT DSIF would be attributed to the absence of the alpha helix, we first screened these mutants for Pol II binding and NELF recruitment. We found that all three were able to bind the Pol II elongation complex (Figs. 5, *B*–*E* and S2, *D*–*F*), When we added NELF to the Pol II-NGN mutant complexes, we further found that all three helix replacement mutants were able to facilitate NELF binding (Fig. 5*D*, lanes 14–15, Figure 5*E*, lanes 10–11 Fig. S3, *B*, *C*, and *E*). The degree of NELF recruitment was comparable to that observed with WT DSIF (Fig. S4, *B*, *C*, and *E*). NELF recruitment was evaluated by comparing lanes in which DSIF was added in saturating amounts (Figs. 5*D* and S3*B* lanes 7 and 15, Figure 5*E*, lanes 7 and 11, S3*C*, lanes 7, 11, and 15, and Fig. S3*E*, lanes 6 and 14).

**Figure 6:**
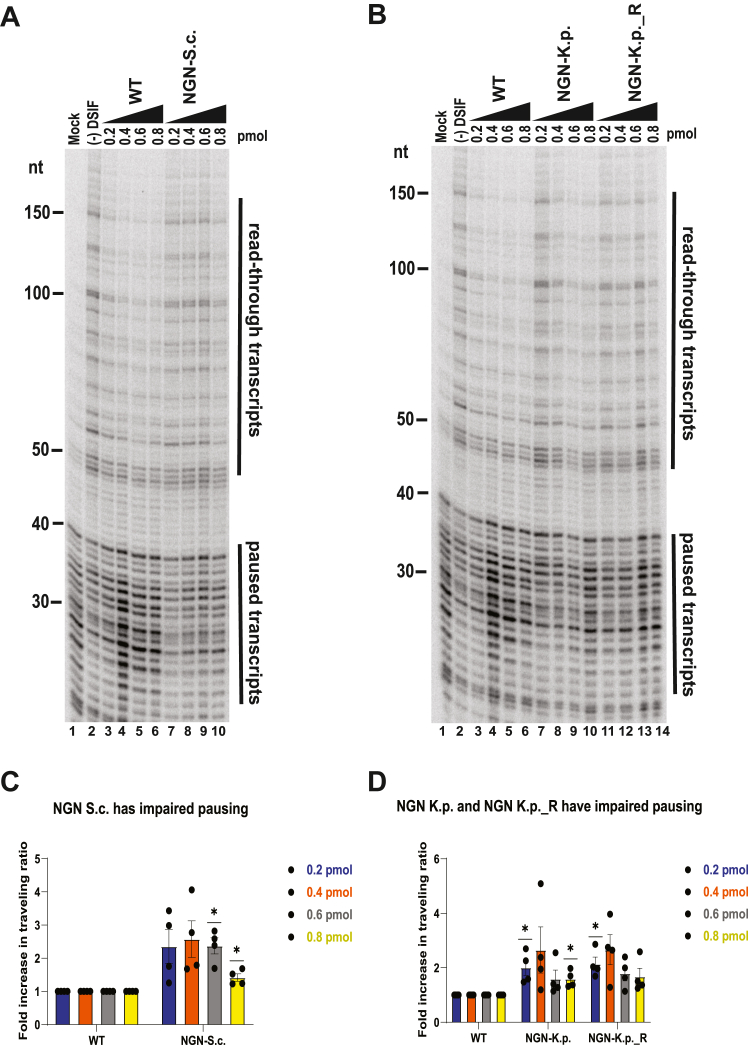
**NGN helix replacement mutants have impaired pausing activity in nuclear extract.** Transcription reactions were set up as in Fig. 4 using DSIF-depleted *Drosophila* nuclear extract with mock-depleted extract as a control. *A*, pausing activity of NGN-S.c. mutant. *B*, pausing activity of NGN-K.p. and NGN-K.p._R mutants. *C*, average fold increase in traveling ratio for NGN-S.c. mutant (*panel A*). Additional replicates can be found in Fig. S6 (n = 4). One star indicates *p* < 0.05. Error bars indicate the standard error of the mean. *D*, average fold increase in traveling ratio for NGN-K.p. and NGN-K.p._R mutants (*panel B*). Additional replicates can be found in Fig. S6 (n = 4). One star indicates *p* < 0.05. Error bars indicate the standard error of the mean.

We then tested the ability of NGN-K.p., NGN-S.c., and NGN-C.e. to restore promoter proximal pausing in *Drosophila* nuclear extract depleted of WT DSIF. All three helix replacement mutants exhibited impaired pausing relative to WT DSIF (Figs. 6 and S6, *A*–*H*). The NGN-C.e. and NGN-S.c. mutants exhibited the greatest degree of pausing impairment, with as much as a ∼2-fold increase in traveling ratio (n = 4, *p* < 0.05) relative to WT DSIF (Fig. 6*A*, cf lanes 7–10 to lanes 3–6, Fig. 6*C* and S6*I*). The NGN-K.p. pausing activity was also impaired, though to a lesser extent than that of the NGN-C.e. and NGN-S.c. mutants (Fig. 6*B*, cf lanes 7–10 and lanes 3–6, Figs. 6*D* and S6*J*). This indicates that the short alpha-helical motif conserved in the NGN domain of NELF-encoding species facilitates promoter proximal pausing.

Since R246 (dR283) is located in the middle of the conserved alpha-helical motif and is oriented toward the non-transcribed template DNA in the structure of the human paused elongation complex (24), we hypothesized that the alpha helix might promote pausing through this residue, possibly by orienting the arginine to interact with the DNA. To test this hypothesis, we generated an NGN-K.p. mutant with a re-inserted R246(dR283) (NGN-K.p._R). We chose to modify the *K. pastoris* sequence because it lacks any deletions in the substituted region, allowing a direct re-insertion of the basic residue (Fig. 5*A*). We predicted that if the positive charge of the arginine side chain is the primary driver in facilitating the pause, the NGN-K.p._R mutant would have to pause activity similar to that of WT DSIF. We further predicted that if the entire helical motif is required to facilitate pausing, re-introducing dR283 would not be sufficient to restore pausing to WT levels.

We first performed EMSAs to confirm that the NGN-K.p._R mutant is able to bind Pol II and recruit NELF (Figs. 5*F*, S2*G*, S3*D*, and S4*D*). We then tested the mutant in our nuclear extract pausing assay. We found that the NGN-K.p._R mutant fails to restore pausing to WT DSIF levels and, in fact, has a pausing defect comparable to that observed with the NGN-K.p. mutant (Fig. 6*B*, cf lanes 11–14 with lanes 7–10, Figs. 6*D* and S6*J*). Thus, dR283 is not sufficient to facilitate promoter proximal pausing in the absence of the rest of the helical motif, indicating that the sequence conserved in NELF-encoding eukaryotes is necessary to promote pausing.

### Identification of DSIF domains involved in NELF recruitment

**Figure 7:**
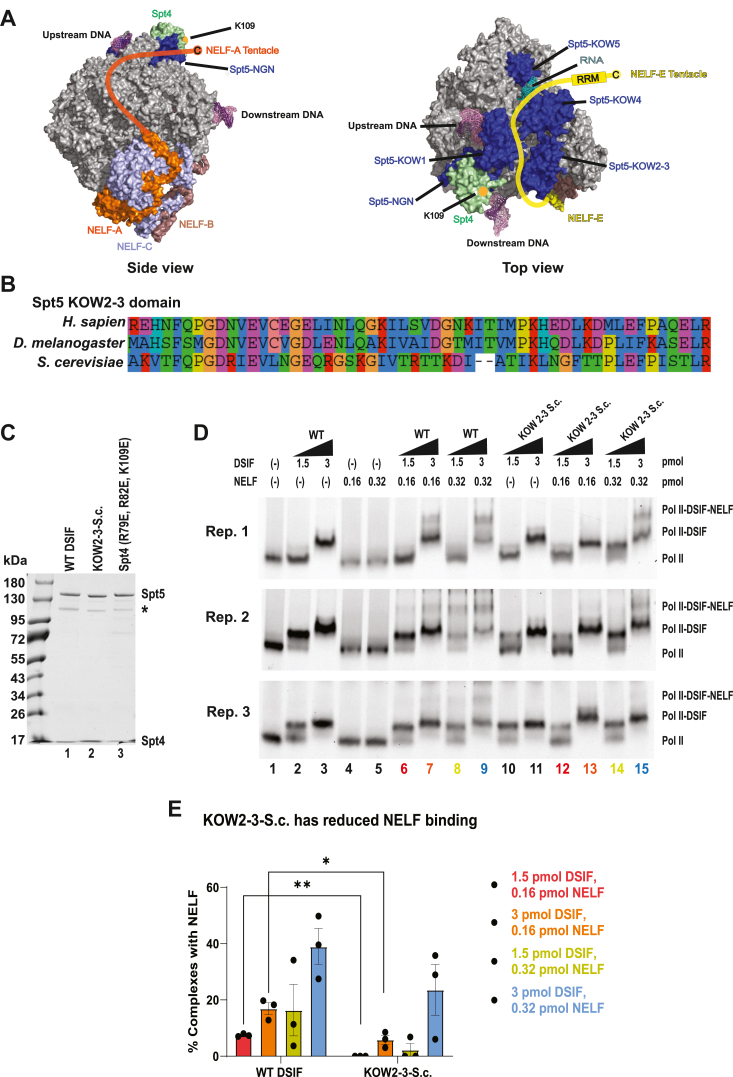
**The KOW2-3 domain of Spt5 is involved in NELF recruitment.***A*, side view (*left*) and top view (*right*) surface representation of paused Pol II-DSIF-NELF complex structure. Modeled paths of NELF-A and NELF-E tentacles are drawn in orange and yellow respectively (24). The conserved Spt4 K109 residue, which is purported to interact with NELF-A, is marked with an orange dot. PDBID: 6GML. *B*, KOW2-3 domain region of Spt5 that was replaced with homologous residues from *Saccharomyces cerevisiae* (human AA 416–469, *Drosophila* AA 457–520, *S. cerevisiae* AA 530–581). *C*, ∼200 ng of WT DSIF, Spt4 (R79E, R82E, K109E), and KOW2-3-S.c. 3 to 14% Tris-Acetate gradient gel, Coomassie-stained. A ∼100 kDa Spt5 degradation product lacking the acidic N-terminal region appears in each preparation (*asterisk*) (30). *D*, Pol II binding and NELF recruitment of KOW2-3-S.c. Stalled elongation complexes were generated as in Figure 3. *E*, quantification of KOW2-3-S.c. NELF recruitment. One star indicates *p* < 0.05 and two stars indicate *p* < 0.01.

Although it has been clear for more than a decade that DSIF plays an indispensable role in the recruitment of NELF to the Pol II elongation complex, how DSIF facilitates this interaction remains unclear. Crosslinking coupled with mass spectrometry experiments have shown that the unstructured C-terminal domain “tentacles” of NELF-A and NELF-E may facilitate NELF association with the Pol II-DSIF complex. NELF-E makes several contacts with the KOW2-3 domain of Spt5 as well as with the KOW4 domain along the mouth of the RNA exit channel. The NELF-A tentacle, in contrast, primarily interacts with Pol II and makes limited contact with Spt4 (Fig. 7*A*) (24). The crosslinker used in these experiments, bis(sulfosuccinimidyl)suberate (BS3) is lysine-specific and has a long spacer arm of 11.4 Å (38), so it is unclear whether the DSIF-NELF contacts identified are bona fide interactions or fortuitous due to the flexible nature of the NELF-A and NELF-E tentacles. We used the crosslinking information to design Spt4 and Spt5 mutants that we hypothesized would disrupt the DSIF-NELF interaction.

We first attempted to generate Spt4 mutants in which all or part of the *Drosophila* Spt4 subunit was replaced with homologous sequences from *S. cerevisiae.* However, this led to the dissociation of the two subunits of the DSIF complex. We then focused on the Spt4 residues that were identified as potential NELF-A contacts by Cramer and colleagues: K81 and K109. Of these two residues, only K109 is conserved in *Drosophila*. We mutated K109 (dK109) to glutamic acid. We also inserted glutamic acid in place of two other residues, N79 (dR79) and P82 (dR82), which have their side chains oriented toward the modeled trajectory of the NELF-A tentacle (Figs. 7*A*, left, and S7*A*). These residues have positive charges in *Drosophila* Spt4 and we hypothesized that the NELF-A-Spt4 interaction could be driven in part by electrostatic interactions with negatively-charged residues in the NELF-A tentacle.

We expressed the Spt4 (R79E, R82E, K109E) DSIF mutant in *E. coli* and purified it as previously described (Fig. 7*C*). We then used EMSA to test the ability of the Spt4 mutant to bind Pol II and recruit NELF. We found that Spt4 (R79E, R82E, K109E) is able to bind Pol II and recruit NELF to a degree comparable to WT DSIF (Fig. S7*B*), indicating that the conserved K109 residue and the nearby basic residues (dR79 and dR82) are not necessary for NELF recruitment.

We next turned our attention to the interaction between Spt5 and the NELF-E subunit. Previous experiments by the Cramer group showed that deletion of the C-terminal tentacle of the NELF-E subunit, which interacts primarily with the KOW4 domain of Spt5, had no effect on Pol II pausing *in vitro*, so we elected to focus on the KOW2-3 domain of Spt5, which has extensive crosslinks to the N-terminal region of NELF-E^24^. To test whether the KOW2-3 region of Spt5 is required for NELF recruitment to the elongation complex, we replaced residues 416 to 469 (d457–510) of Spt5 with the homologous region from *S. cerevisiae* to generate a KOW2-3-S.c. mutant. This region is well conserved from human to *Drosophila* but diverges in yeast (Fig. 7*B*). We expressed and purified this mutant from *E. coli* (Fig. 7*C*). We then used EMSA to test its Pol II binding and, crucially, to determine whether the mutant is able to recruit NELF. We found that the KOW2-3-S.c. mutant binds Pol II (Fig. 7*D*, cf lanes 3 and 11). We further observed that at 0.32 pmol of NELF added, the KOW2-3-S.c. mutant was able to recruit NELF to the Pol II-DSIF complex (Fig. 7*D*, cf lanes 9 and 15, Fig. 7*E*). However, lowering the quantity of NELF in the reaction to 0.16 pmol resulted in reduced binding between the Pol II-KOW2-3-S.c. complex and NELF relative to WT DSIF (Fig. 7*D*, Fig., cf lanes 6 and 7–12 and 13, Fig. 7*E*, red and orange bars). This difference was statistically significant (Fig. 7*E*, *p* < 0.05 for 3 pmol DSIF and *p* < 0.01 for 1.5 pmol DSIF). Furthermore, the KOW2-3-S.c. mutant was only able to recruit NELF when higher quantities of both the KOW2-3-S.c. mutant and NELF were added and did so to a lesser degree than WT DSIF (Fig. 7*D*, cf lanes 9 and 15, Fig. 7*E*, compare data points on blue bars). In contrast, the Pol II-DSIF-NELF supershift was observed with WT DSIF even at 1.5 pmol of DSIF, a quantity that was not sufficient to generate a full mobility shift of the Pol II elongation complex (Fig. 7*D*, cf lanes 6 and 8 with lane 12 and 14). These results indicate that the KOW2-3 domain of Spt5 is involved in DSIF’s recruitment of NELF to the elongation complex.

### Expression of Spt5 mutants in *Drosophila*

**Figure 8:**
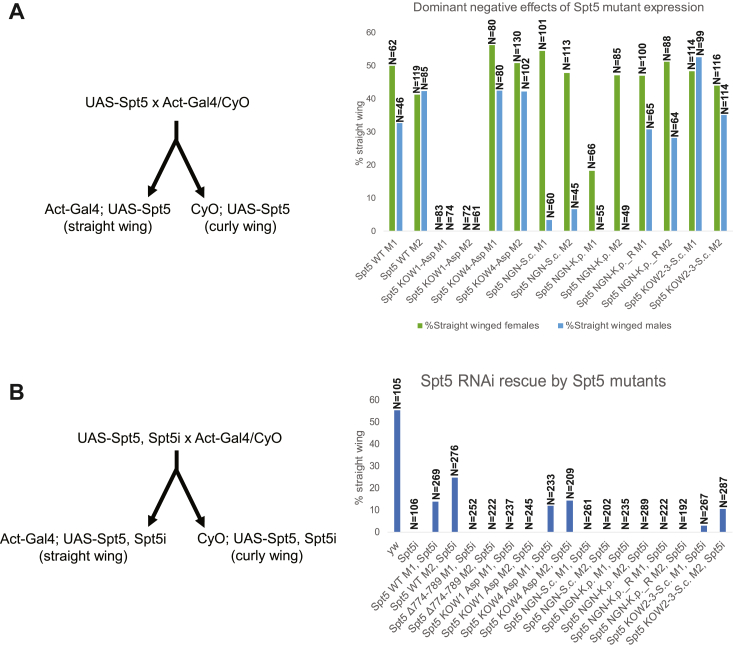
**Lethality caused by ectopic expression of Spt5 mutants in *Drosophila***. *A*, UAS-Spt5 mutant females were mated to Act-Gal4/CyO males to generage Act-Gal4; UAS-Spt5 (*straight wing*) and CyO; UAS-Spt5 (*curly wing*) flies. Progeny were scored and the percentage of straight winged males and females was calculated. Numbers presented above each bar (N=) are the sum of curly and straight wing flies counted. M1 and M2 represent two independent lines. Straight winged flies express both the endogenous WT Spt5 as well as the ectopic mutant Spt5. Expression of mutant Spt5 in straight-winged adults was confirmed by western blotting (Fig. S8). *B*, UAS-Spt5, Spt5i mutants were mated to Act-Gal4/CyO flies to generage Act-Gal4; UAS-Spt5, Spt5i (*straight wing*) and CyO; UAS-Spt5, Spt5i (*curly wing*) flies. Progeny was scored and the percentage of straight-winged flies was calculated. M1 and M2 represent two independent lines. Straight-winged flies express the mutant Spt5; expression of the endogenous Spt5 is knocked down by RNAi. Matings of Act-Gal4/CyO to yw and Spt5i were set up as positive and negative controls, respectively. Spt5 Δ774 to 789, Spt5i was mated to Act-Gal4/CyO as an additional negative control because our group has shown previously that this mutant does not rescue the lethality conferred by the Spt5 RNAi (30).

We next tested the effects of our Spt5 mutations *in vivo*. To do this, we generated transgenic flies expressing the KOW1-Asp, KOW4-Asp, NGN-S.c., NGN-K.p., NGN-K.p._R, or KOW2-3-S.c. mutants under the control of a Gal4 upstream activating sequence. We first determined whether expression of any of these Spt5 mutants would result in lethality if expressed in the presence of the endogenous WT Spt5 as this would indicate that the mutant was somehow interfering with the function of the WT Spt5. We mated UAS-Spt5 flies to actin-Gal4/CyO flies, which ubiquitously express Gal4 throughout development. Of the progeny, the flies that carry Gal4 and express transgenic Spt5 have straight wings while flies that carry CyO and do not express transgenic Spt5 have curly wings. We calculated the percentage of straight-winged male and female progeny for each mutant (Fig. 8*A*).

We found that expression of KOW1-Asp was lethal in males and females, and expression of the NGN mutants resulted in a male-specific lethality. Expression of NGN-S.c. resulted in a drastic reduction in the percentage of male straight-winged flies while expression of the NGN-K.p. mutant resulted in zero straight-winged males. Surprisingly, while we saw no difference between the NGN-K.p. and NGN-K.p._R mutants in our nuclear extract pausing assay, there was a notable difference *in vivo.* Expression of NGN-K.p._R diminished the level of male-specific lethality observed in NGN-K.p.-expressing flies, suggesting the conserved dR283 is critical to the function of the NGN domain (Fig. 8*A*).

In contrast to the aforementioned mutants, expression of the KOW4-Asp and KOW2-3-S.c. mutants caused no lethality. The percentage of straight-winged flies and the ratio of males to females was comparable to the transgenic expression of WT Spt5. To assess the possibility that the absence of lethality might be due to an absence of expression, we determined if the mutant proteins were being expressed by monitoring the expression of the FLAG-tagged derivatives of Spt5 in the heads of the straight-winged flies. Western blotting with the anti-FLAG antibody showed that the KOW4-Asp and KOW2-3-S.c. mutants as well as female NGN mutants expressed FLAG-tagged derivatives of Spt5 at levels comparable to the WT FLAG-tagged Spt5 (Fig. S8). Hence, these mutants were being expressed but not interfering with the function of the endogenous wild-type Spt5.

We were unable to determine directly the level of mutant Spt5 expressed relative to the endogenous WT Spt5 because of their similarities in size. However, we obtained an estimate by comparing the level of an Spt5 deletion mutant, Δ635 to 789, to endogenous Spt5 with an Spt5 antibody and then comparing the level of the FLAG-tagged Δ635 to 789 mutant to the other FLAG-tagged derivatives detected with the anti-FLAG antibody (Fig. S8). We estimate that the ectopic derivatives were expressed at two to three-fold lower levels than the endogenous Spt5 in fly heads.

We then wanted to see whether the expression of the Spt5 mutants *in vivo* would support fly viability if the endogenous Spt5 was depleted by RNAi. To test this, we recombined the Gal4-regulated Spt5 transgenes with a Gal4-regulated RNAi to generate UAS-Spt5, Spt5i flies. The transgenes encoding the ectopic Spt5 contain synonymous mutations that confer resistance to the Spt5 RNAi (30). We mated UAS-Spt5, Spt5i flies to actin-Gal4/CyO flies and scored the number of straight-winged and curly-winged flies. Straight-winged flies carry actin-Gal4 and express both the Spt5 RNAi and the Spt5 transgene. We calculated the percentage of straight-winged progeny for each mutant (Fig. 8*B*).

We found that ectopic expression of the KOW4-Asp and KOW2-3-S.c. mutants was able to support fly viability in the presence of Spt5 RNAi. As in our dominant negative experiment, expression of the KOW1-Asp mutant failed to support viability. We also found that the NGN-S.c., NGN-K.p., and NGN-K.p._R mutants were unable to rescue fly viability upon Spt5 RNAi knockdown. This was not simply due to the lethality of the NGN mutant protein since expression of the NGN mutants in the absence of the Spt5 RNAi yielded females (Fig. 8*A*). Therefore, the NGN helical motif is required for fly development (Fig. 8*B*).

## Discussion

### The role of the KOW4 domain in pausing

The work described herein provides a functional assessment of the roles of various Spt5 domains in facilitating promoter proximal pausing. Here, we have shown that, in addition to mediating interactions between NELF and the Pol II elongation complex, DSIF facilitates promoter proximal pausing through the KOW4 and NGN domains of the Spt5 subunit. The KOW4 domain interacts extensively with the nascent transcript; work from the Cramer group has shown that this domain switches from a “closed” to an “open” conformation when the elongation complex transitions from a paused state to an active state (24), suggesting that disengagement of the KOW4 domain from the RNA is a prerequisite for pause release. Our work herein supports this hypothesis. We have shown that reversing the charge of KOW4 residues anticipated to interact with the RNA results in a pausing defect in *Drosophila* nuclear extract (Fig. 4, *D* and *G*). Notably, this defect is accompanied by robust Pol II binding and NELF recruitment that is comparable to that of WT DSIF, indicating an effect of our mutations on Pol II pausing (Figs. 3, *D* and *F* and S4*E*). Thus, the maintenance of the promoter proximal pause is likely dependent in part on the KOW4-RNA interaction, which is likely disrupted by the opening of the Spt5 RNA clamp.

The KOW4 domain’s interaction with the nascent transcript may depend on the phosphorylation state of the linker region between the KOW4 and KOW5 domains. Work from the Fisher and Adelman labs has shown that phosphorylation of this region by P-TEFb can act as a switch that determines whether Pol II enters productive elongation or prematurely terminates (39, 40). Phosphorylation of the KOW4-5 linker on Ser666 by P-TEFb in human cells is associated with an increased proportion of Pol II in the gene body (39). This phosphorylation event may result in structuring of the flexible linker that forces the opening of the RNA clamp, allowing pause release. The KOW4–RNA interaction may also be mediated by NELF-E. The flexible NELF-E tentacle was shown to crosslink to the Spt5-KOW4 domain along the mouth of the RNA exit channel (24). Interaction with NELF may help stabilize the KOW4 domain in the “closed” position, facilitating pausing.

Ectopic expression of the KOW4-Asp mutant in *Drosophila* did not have a dominant negative effect and the mutant was able to support viability in flies expressing Spt5 RNAi (Fig. 8). This suggests that mutating the RNA-interacting residues of the KOW4 domain may not be sufficient to fully disrupt the promoter proximal pause *in vivo*. Additional contacts provided by the Spt5 NGN domain, NELF, and other factors such as nucleosomes (23) present a much more complex regulatory context than the one we reconstituted using *Drosophila* nuclear extract, which could account for the apparent discrepancy between our *in vitro* and *in vivo* results.

### The role of the NGN domain in pausing and Pol II processivity

The Spt5 NGN domain also plays a significant role in pausing. We have shown that replacement of a short helical motif in the *Drosophila* NGN domain with homologous unstructured loop regions from yeast or worms results in a severe pausing defect (Fig. 6) while leaving Pol II binding and NELF recruitment functions intact (Fig. 5). This is the first report of a role for the NGN domain in transcriptional pausing in a eukaryotic system.

We explored the possible function of a conserved arginine, hR246(dR283), that was oriented to interact with the non-transcribed template strand. Though we did not observe a difference in pausing activity between our NGN-K.p. and NGN-K.p._R mutants in nuclear extract, re-insertion of the arginine had a dramatic effect in flies. The NGN-K.p._R mutant had a less severe dominant negative effect than its NGN-K.p. counterpart, indicating that the conserved arginine residue is critical to the NGN domain’s function (Fig. 8*A*). Neither the NGN-S.c., NGN-K.p., nor the NGN-K.p._R mutants were able to support *Drosophila* viability when expressed in the presence of Spt5 RNAi, indicating that the full NGN alpha-helical motif is necessary for proper fly development (Fig. 8*B*).

**Figure 9:**
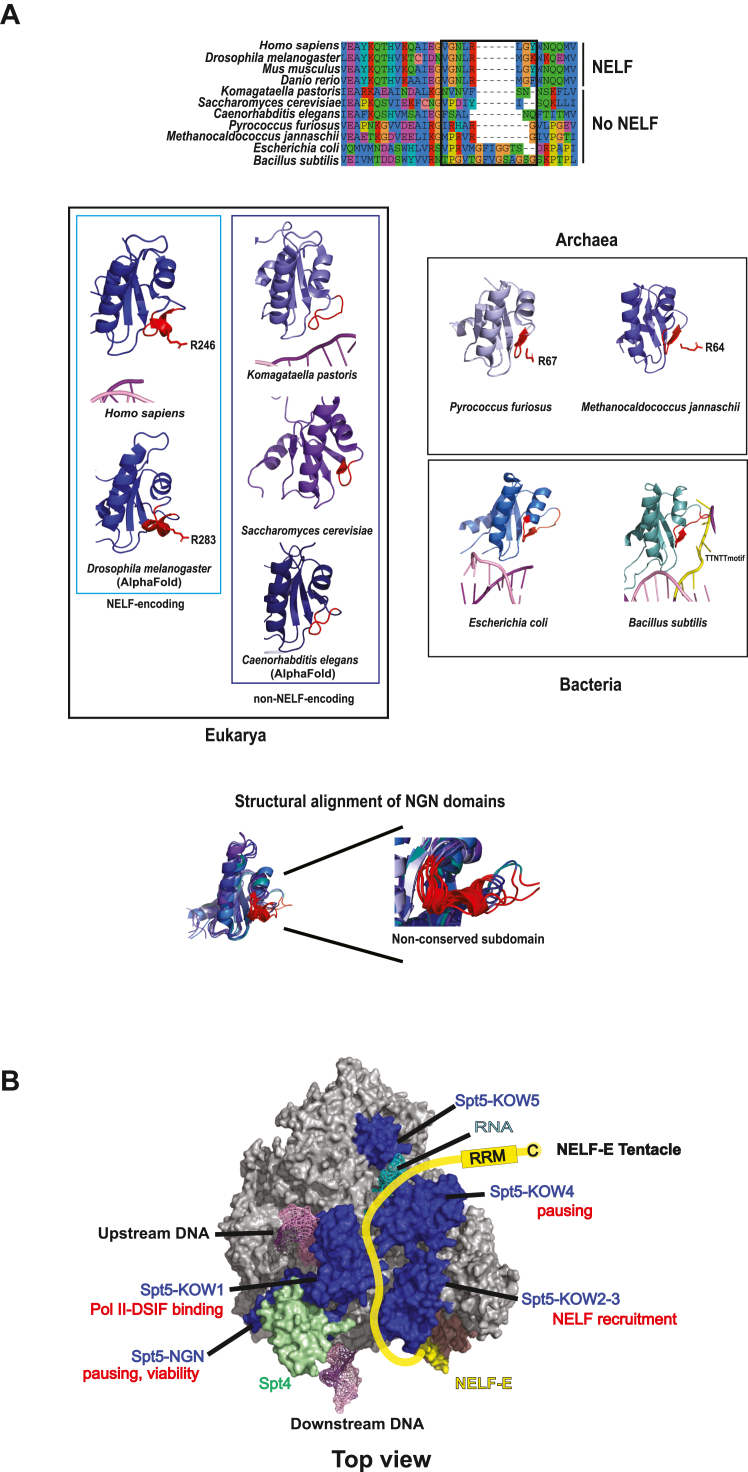
**Summary of Spt5 function**. *A*, NGN domains are structurally conserved across the domains of life. A subdomain (*black box*, *red*) diverges and may be responsible for distinct functions in different organisms. An alpha helical motif in the NGN domain is conserved in NELF-encoding species but not in species without NELF (*black box*, *top*). The arginine contained in this alpha helix is also conserved in archaea, but is located in a beta strand. PDB ID: 6GML, 5XOG, 2EXU, 3P8B, 3LPE, 6C6U. Structures for *Drosophila* and *C. elegans* were modeled using Alphafold (65, 66). Structural alignment (*bottom*) of the NGN domains indicates a high degree of structural conservation between species; the subdomain (colored in *red*) exhibits a degree of structural variability not observed in other parts of the NGN domain. *B*, our biochemical analyses reveal functions of different domains of Spt5. The NGN and KOW4 domains facilitate promoter proximal pausing, while the KOW1 and KOW2-3 domains mediate binding to the Pol II elongation complex and NELF recruitment respectively.

Experiments in *Bacillus subtilis* previously described RNA polymerase pausing mediated by the interaction of the NGN domain of the bacterial homolog NusG with the non-transcribed DNA in the transcription bubble. However, unlike in *Drosophila*, this process is dependent on the presence of a DNA sequence motif and does not involve a helical motif similar to what we describe here (41, 42, 43). Indeed, the alpha helical motif appears to be exclusive to NELF-encoding eukaryotes, though the conserved hR246 (dR283) residue also appears in archaeal species. Available structures of archaeal Spt5 indicate that this arginine is located in a beta strand rather than the alpha helix found in metazoans (44, 45). This beta strand is also present in *E. coli* and in *B. subtilis*, but both these species lack the conserved arginine found in NELF-encoding eukaryotes and archaea (Fig. 9*A*). Notably, in archaea, the NGN domain is required for stimulation of elongation (44), suggesting that the function of the conserved arginine is context-dependent.

The NGN domain is highly conserved across all domains of life and exhibits significant structural similarity from species to species (Fig. *A*). Paradoxically, the function of this domain is varied. In some cases, such as *E. coli,* archaea, and *S. cerevisiae,* the NGN domain stimulates elongation (44, 46, 47, 48), but in *B. subtilis* and *Drosophila*, the NGN domain promotes pausing (41, 49). We propose that the DNA-interacting region of the NGN domain is a subdomain that has evolved to serve different functions in various species. This may explain how the highly conserved NGN domain can serve as both a stimulator and a repressor of transcription.

Ectopic expression of the NGN-S.c. and NGN-K.p. mutants greatly inhibited the development of adult male flies (Fig. 8*A*). In *Drosophila*, the NGN domain may also promote dosage compensation by stimulating the upregulation of genes on the single male X chromosome. Spt5 has been shown to interact with the dosage compensation factor male-specific lethal (MSL1) through the NGN domain as well as through the KOW domains (50). Though the mechanisms of this interaction are unknown, it is possible that our mutations of the NGN domain disrupted either the association between Spt5 and MSL1 or their joint function, resulting in the male-specific dominant negative effect we observed (Fig. 8*A*). The NGN domain’s non-transcribed-DNA-interacting region is likely a hotspot for regulating Pol II processivity, making it a logical target for transcription regulation by MSL1.

Our NGN mutations may have also disrupted the function of RNA polymerase I (Pol I). Mass spectrometry and immunoprecipitation experiments in yeast demonstrated that Pol I is able to associate with Spt4/5 and later genetic studies demonstrated that Spt5 regulates Pol I transcription (51, 52). This interaction is mediated at least in part by the Spt5 NGN domain (53). Thus, it is possible that replacing the NGN helical motif *in vivo* disrupted not only the processivity of Pol II but also the processivity of Pol I, dysregulating the synthesis of ribosomal RNA. Such a substantial disruption would account not only for the failure of the NGN mutants to support *Drosophila* viability but could also explain the dominant lethality of the KOW1-Asp mutant given that the KOW1 and NGN domains together form the DNA clamp.

### Mechanisms of DSIF binding to the elongation complex

We also demonstrated that disrupting the interaction between the Spt5 KOW1 domain and the upstream DNA results in impaired binding of DSIF to the Pol II elongation complex (Fig. 3*B*). This is in agreement with previous work in yeast, which showed that deletion of this domain reduced the affinity of Spt5 for the elongation complex (48). The KOW1 domain is the only KOW domain conserved across all three domains of life (54), so its role in Pol II elongation complex binding is likely a conserved feature in Spt5 and Spt5 homologs. Ectopic expression of the KOW1-Asp mutant in *Drosophila* had a dominant lethal effect, highlighting the importance of this region. In addition to facilitating Spt5-Pol II interaction, the KOW1 domain also ensures physical separation of the upstream DNA and the transcript (24, 29, 55), potentially preventing the formation of irregular structures such as R-loops (56), which have been linked to genome instability (57).

Surprisingly, we observed no elongation complex binding defect in the KOW4-Asp mutant (Fig. 3*D*), suggesting that the interaction between the KOW4 domain and the nascent transcript is not necessary for DSIF-Pol II binding. This is in contrast to previous studies in yeast and *Drosophila*. In yeast, digestion of the nascent transcript with RNaseI nearly eliminated Spt5 binding to the elongation complex (48). Moreover, a prior study from our group showed that DSIF failed to bind to Pol II elongation complexes that had transcripts shorter than 18 nucleotides (19). However, because varying the transcript length in these elongation complexes also resulted in varying the length of the upstream DNA, the decrease in DSIF binding could be attributed to reduced interaction between the DNA template and the Spt5 KOW1 domain rather than loss of the KOW4–transcipt interaction. Complexes with 18 nt transcripts only have ∼4 base pairs of double-stranded upstream DNA extending out of the Pol II; based on the structures of the human elongation complex, association with the KOW1 domain requires at least ∼10 bp of upstream DNA (24, 28).

The mechanism of Pol II-DSIF association may nevertheless rely on multiple contact points. While we have shown that the KOW1 domain is necessary for initial Pol II elongation complex-DSIF binding, recent structural experiments from the Farnung lab demonstrated that Spt5 can be retained on the elongation complex despite the displacement of the KOW1 and NGN domains and Spt4 (58). This suggests that the RNA clamp formed by the KOW4 and KOW5 domains may function to preserve the Pol II-DSIF interaction after the initial association. Furthermore, though we saw no effect of our Spt5 KOW2-3 domain mutations on Pol II binding, it is possible that this region also plays a stabilizing role that helps maintain the association of DSIF with the elongation complex during various conformational transitions.

### Mechanism of NELF recruitment to the elongation complex

Of the nine DSIF mutants described here, all but one were able to bind NELF to a degree that was comparable to WT DSIF. This is perhaps unsurprising since the mutations in the Spt5 NGN and KOW1 domains are not located near the modeled paths of the NELF-A and NELF-E tentacles (Fig. 7*A*, note the DNA-interacting faces of the NGN and KOW1 domains and their lack of proximity to NELF). Moreover, we observed no effect on NELF binding by the mutations in the Spt5 KOW4 domain (Fig. 3*F*). The NELF-E C-terminal tentacle is thought to stretch across the mouth of the RNA exit channel between the nascent transcript and the KOW4 domain, so it seemed likely that disrupting the contact between the Spt5 domain and the RNA would also disturb the NELF-E interaction. Nevertheless, our observation is in line with that of the Cramer group, who deleted the NELF-E tentacle and failed to see an effect on pausing *in vitro* (24). Furthermore, mutating a pocket of residues (Spt4 R79, R82, K109) in close proximity to a putative contact point between Spt4 and NELF-A that was previously identified by crosslinking mass spectrometry had no effect on NELF recruitment, suggesting that crosslinking results must be interpreted with caution and followed up with biochemical analyses, particularly with regards to intrinsically disordered regions such as the NELF-A tentacle. It is possible that NELF recruitment is mediated in part by Spt4-NELF-A interaction, but verifying this will require careful and systematic biochemical assessment of both Spt4 and the NELF-A C-terminus. Biochemical data from the Cramer lab suggests that deletion of the NELF-A tentacle impairs Pol II pausing *in vitro* (24), so future work to interrogate the intrinsically disordered regions of this subunit will be necessary for a complete mechanistic description of NELF recruitment to the elongation complex.

Mutating the KOW2-3 domain of Spt5 reduced NELF binding in our *in vitro* system (Fig. 7, *D* and *E*). The KOW2-3 domain is located near the modeled path of the NELF-E N-terminal region and has the greatest number of putative NELF-E contacts (24). Notably, NELF binding was not completely abolished and could be restored by adding greater quantities of NELF. Moreover, the KOW2-3-S.c. mutant exhibited no dominant negative effect when expressed in flies and was able to rescue the effects of RNAi knockdown of endogenous Spt5 (Fig. 8), suggesting that while the KOW2-3 domain contributes for NELF recruitment to the elongation complex, the mutations we made did not interfere with development. Recent work in human cells showed that the formation of biomolecular condensates mediated by the NELF-A tentacle enhances the recruitment of NELF to promoters (59). It is possible a similar phenomenon occurs at *Drosophila* promoters, resulting in a cellular concentration of NELF that is sufficient to overcome the defect of the KOW2-3-S.c. mutant. Interactions between NELF and Spt4, NELF and Pol II, as well as NELF and the nucleic acid scaffold likely serve as additional stabilizing contact points and may even drive the initial recruitment of the NELF complex.

### Conclusion and future directions

We performed an extensive analysis of the domains of the larger DSIF subunit, Spt5, and showed that the NGN and KOW4 domains facilitate pausing in a manner distinct from the role of DSIF as the mediator of NELF-Pol II interaction. We also showed that the KOW1 domain facilitates DSIF binding to the Pol II elongation complex and that the KOW2-3 domain contributes to NELF recruitment (Fig. 9*B* and Table 1).

**Table 1:** Summary of DSIF mutants

Mutant	Pol II EC binding	NELF recruitment	Pausing in nuclear extract	Dominant negative effect *in vivo*	Supports viability
KOW1-Asp	Decreased	Yes	Decreased	Lethal	No
KOW4-Asp	No change	Yes	Decreased	None	Yes
NGN-Asp	No change	Yes	No change	—	—
NGN-S.c.	No change	Yes	Decreased	Male specific	No
NGN-C.e.	No change	Yes	Decreased	—	No
NGN-K.p.	No change	Yes	Decreased	Male specific	No
NGN-K.p._R	No change	Yes	Decreased	Male specific, less severe than NGN-K.p.	No
KOW2-3-S.c.	No change	Decreased	—	None	Yes
Spt4 (R79E, R82E, K109E)	No change	No change	—	—	—

The role of the NGN domain in particular warrants further investigation. We identified a modular subdomain that is in close proximity to the non-transcribed template DNA strand. This subdomain has broad amino acid and structural divergence among the three domains of life despite the high degree of structural *conservation* in the rest of the NGN domain (Fig. 9*A*). The modular region could account for the various reported functions of the NGN domains present in Spt5 and Spt5 homologs.

## Experimental procedures

### Generation of nuclear extract

Nuclear extracts for *in vitro* transcription assays and for purifying Pol II were made from 0∼14 h *Drosophila* embryos as described previously with modifications (30, 32, 33). Embryos were collected from large populations of Oregon R or FLAG-Rpb1 flies at 12 to 14 h intervals and stored at 4 °C for up to 3 days. After 3 days, embryos were dechorionated and suspended in 3 ml of Buffer A for each gram of embryos. Buffer A consisted of 15 mM HEPES, pH 7.6, 10 mM KCl, 5 mM MgCl_2_, 0.1 mM EDTA, 0.5 mM EGTA, and 350 mM sucrose and was freshly supplemented with 1 mM DTT, 1 mM sodium bisulfite, 0.2 mM PMSF, 1.6 μg/ml benzamidine-HCl, 1 μg/ml aprotinin, 1 μg/ml pepstatin A, and1 μg/ml leupeptin prior to use. The suspension was homogenized with five strokes of a Potter Elvehjem tissue homogenizer and strained through one layer of Miracloth. After most of the lysate was filtered, the retentate was rinsed with an additional 3 ml of Buffer A, resulting in a twofold dilution of the lysate. The lysate was distributed evenly among 200 ml centrifuge bottles and centrifuged in a precooled SLA-1500 rotor at 9000 rpm for 15 min to collect the nuclei. The supernatant was slowly decanted and the pellet was resuspended in 1 ml of Buffer AB (15 mM HEPES, pH 7.6, 110 mM KCl, 5 mM MgCl_2_, 0.1 mM EDTA with freshly added 1 mM DTT, 1 mM sodium bisulfite, 0.2 mM PMSF, 1.6 μg/ml benzamidine-HCl, 1 μg/ml aprotinin, 1 μg/ml pepstatin A, and 1 μg/ml leupeptin) per gram of embryos. The nuclei were fully suspended using the tight pestle of a Dounce homogenizer and the suspension was distributed equally among polycarbonate ultracentrifuge tubes. 1/10 volume of 4 M ammonium sulfate pH 7.9 was added to each tube to a final concentration of 0.36 M ammonium sulfate. The tubes were then mounted on a rotating wheel at 4 °C for 15 to 20 min and subsequently ultracentrifuged in a pre-cooled Ti70 rotor at 35,000 rpm for 1 h. This ultracentrifugation step resulted in a gelatinous pellet at the bottom of the tube and a cloudy white lipid layer at the top. The supernatant was collected from each tube by plunging a 10 ml pipette below the lipid layer and pooled into a single graduated cylinder to measure the volume. The material was then transferred to a cold beaker with a stir bar on an ice tray. For every mL of lysate, 0.3 g of finely-ground ammonium sulfate was added to the stirring solution over a period of 5 min. The solution was then left to stir for an additional 10 min. The lysate was subsequently centrifuged in a precooled SS34 rotor at 15,000 rpm for 20 min and the supernatant was decanted. Using a Dounce homogenizer, the pellet was suspended in 0.2 ml Buffer C (25 mM HEPES, pH 7.6, 40 mM KCl, 12.5 mM MgCl_2_, 0.1 mM EDTA, 10% glycerol supplemented with fresh 1 mM DTT, 0.5 mM Na bisulfite, 0.1 mM PMSF, 1.6 μg/ml benzamidine-HCl, 1 μg/ml aprotinin, 1 μg/ml pepstatin A, and1 μg/ml leupeptin) per gram of embryos. The extract was then dialyzed against 2 L of Buffer C in Spectra/Por 1 Dialysis Membrane (6000–8000 MWC) tubing until the conductivity of the sample was equal to 0.15 mM HEMGK (25 mM HEPES, pH 7.6, 0.1 mM EDTA, 12.5 mM MgCl_2_, 10 % glycerol, 0.15 M KCl). After dialysis, the extract was transferred to clean tubes and spun for ∼20 s in a microfuge to remove any precipitate. Extracts were then transferred to fresh tubes, flash-frozen in liquid nitrogen, and stored at −80 °C for subsequent use in transcription reactions or for Pol II purification.

### Purification of Pol II

Pol II was purified using nuclear extract from *Drosophila* embryos containing a FLAG tag on the C-terminal end of the Rpb1 subunit (31). All steps were performed at 4 °C. Nuclear extract was thawed on ice and protease inhibitors were freshly added to the following final concentrations: 0.1 mM PMSF, 1.6 μg/ml benzamidine-HCl, 1 μg/ml aprotinin, 1 μg/ml pepstatin A, and 1 μg/ml leupeptin. A 40 ml POROS Heparin (Thermo Scientific catalog no. 4329437) column pre-equilibrated with 0.15 M HEMGK (25 mM HEPES, pH 7.6, 0.1 mM EDTA, 12.5 mM MgCl_2_, 10 % glycerol, 0.15 M KCl) was loaded with ∼30 ml nuclear extract at a flow rate of 1 ml/min. The column was washed with 5 column volumes (CV) of 0.15 M HEMGK at 1 ml/min and the protein was eluted with 5 CV of 0.4 M HEMGK (25 mM HEPES, pH 7.6, 0.1 mM EDTA, 12.5 mM MgCl_2_, 10 % glycerol, 0.4 M KCl) and collected in 10 ml fractions. Fractions were assayed for Pol II using a promoter-independent transcription assay (described below). Peak fractions were pooled for the next purification step.

Anti-FLAG M2 resin (Sigma Aldrich catalog no. A2220) was prepared by washing it with 0.1 M glycine HCl, pH 3.5 according to the manufacturer's instructions, and equilibrated with 0.5 M HEMGK (25 mM HEPES, pH 7.6, 0.1 mM EDTA, 12.5 mM MgCl_2_, 10 % glycerol, 0.5 M KCl). In general, 0.5 ml of packed resin is sufficient for a Pol II preparation that uses 30 ml of nuclear extract derived from approximately 150 g of embryos. The pooled sample from the Heparin column was mixed with protease inhibitors (0.1 mM PMSF, 1.6 μg/ml benzamidine-HCl, 1 μg/ml aprotinin, 1 μg/ml pepstatin A, and1 μg/ml leupeptin) and incubated in bulk with the pre-equilibrated Anti-FLAG M2 resin at 4 °C on a turning wheel. After 1.5 h, the suspension was transferred to an empty BioRad gravity flow column and allowed to drain. The column was washed with 10 CV 0.6 M HEMGK (25 mM HEPES, pH 7.6, 0.1 mM EDTA, 12.5 mM MgCl_2_, 10 % glycerol, 0.6 M KCl), then washed with an additional 10 CV of 0.15 M HEMGK. Elution was carried out in 10 0.5CV steps with 0.15 M HEMGK supplemented with 200 μg/uL 3× FLAG peptide. For each elution step, the column was capped and allowed to incubate with the elution buffer prior to draining: 30 min for the first three elutions and 10 min for elutions 4 to 10. Longer incubation times increase the efficiency of the elutions and the concentration of Pol II in early fractions. In general, the first three elution fractions have the greatest amount of Pol II.

### Pol II activity assay

Pol II transcription activity was evaluated using a promoter-independent assay that measured the incorporation of radioactive CTP into nascent transcripts (60, 61). Transcription reactions were performed by mixing 1.35 μl of Pol II fraction with 2.65 μl of the reaction mix. The reaction mix contained 2 uL of 2× transcription mix (2 mM MnCl_2_, 50 mM HEPES pH 7.6, 24% glycerol, 100 mM (NH_4_)_2_SO_4_, 1 mM DTT, 160 μg/ml denatured, sonicated salmon DNA), 0.4 uL of NTP mix (5 mM GTP, 5 mM ATP, 5 mM UTP, 0.01 mM CTP), 0.15 uL of ddH_2_O or 266 μg/ml alpha amanitin, 0.1 μl of [α-^32^P] CTP (6000 Ci/mmol, 10 μCi/μl). Reactions were incubated at room temperature for 20 min. After 20 min, 4 μl of each reaction were spotted onto a 1-cm^2^ piece of DEAE paper to bind transcripts. The DEAE paper was washed three times with ∼10 ml of 5% K_2_HPO_4_, 0.1% Na pyrophosphate. Radioactivity was measured using a scintillation counter.

### Generation of DSIF mutant plasmids

DSIF mutant expression constructs were generated by modifying a previously generated pST44-Spt5-Spt4 expression plasmid (30). gBlocks (Integrated DNA Technologies) containing the appropriate mutations were inserted into digested pST44-Spt5-Spt4 vectors using InFusion (Takara catalog no. 638943). For the NGN mutants, the pST44-Spt5-Spt4 plasmid was digested with ScaI and AflII. To generate the KOW1-Asp mutant, the pST44-Spt5-Spt4 plasmid was digested with ScaI and DraI. For the KOW4-Asp mutant, the pST44-Spt5-Spt4 plasmid was digested with AleI and PsiI. For the KOW2-3 S.c. mutant, the pST44-Spt5-Spt4 plasmid was digested with BglI and AleI. For the Spt4 (R79E, R82E, K109E) mutant, the pST44-Spt5-Spt4 plasmid was digested with HindIII and MluI. Mutant plasmids were verified by Sanger sequencing.

### Expression and purification of recombinant DSIF

Wild-type DSIF and DSIF mutants were expressed in *E. coli* using the polycistronic vector pST44 containing the coding sequences for C-terminal His_6_-tagged Spt4 and C-terminal FLAG-tagged wild-type or mutant Spt5 (30, 34). Expression was carried in Rosetta (DE3) pLysS cells as previously described (30). Proteins were purified as previously described (30) with some modifications. Harvested cells from 1 L of culture were suspended in 40 ml TBS150 (50 mM Tris-HCl pH 7.5, 150 mM NaCl, 10% glycerol) along with protease inhibitors (0.1 mM PMSF, 1.6 μg/ml benzamidine-HCl, 1 μg/ml aprotinin, 1 μg/ml pepstatin A, and1 μg/ml leupeptin). Samples were flash-frozen and thawed for sonication or stored at −80 °C for several weeks. Prior to sonication, fresh protease inhibitors were added to each thawed sample (0.1 mM PMSF, 1.6 μg/ml benzamidine-HCl, 1 μg/ml aprotinin, 1 μg/ml pepstatin A, and 1 μg/ml leupeptin). The cell suspension was sonicated for ∼30 cycles (15 s on, 45 s off, Branson Sonifier 450, Duty Cycle: 50%, Output: 5) or until the solution became noticeably less viscous and then cleared by centrifugation at 20,000*g* for 20 min. Samples were chilled on ice during sonication. The supernatant was incubated with Talon metal affinity resin (Takara catalog no. 635506, 1 ml bed volume per 40 ml lysate) in bulk on a rotating wheel overnight at 4 °C. The resin was collected by centrifugation at 700*g* and washed in bulk at 4 °C for 15 min with 20 CV of TBS300 (50 mM Tris-HCl pH 7.5, 300 mM NaCl, 10 % glycerol). The resin was transferred to a gravity flow column and washed with an additional 10 CV of TBS300 with 5 mM imidazole to reduce non-specific binding. Proteins were eluted in eight 0.5 CV steps with TBS300 containing 200 mM imidazole. Elution fractions were analyzed by SDS-PAGE and Coomassie staining and fractions containing detectable DSIF were pooled for subsequent purification with anti-FLAG resin.

Protease inhibitors were added to the pooled sample (0.1 mM PMSF, 1.6 μg/ml benzamidine-HCl, 1 μg/ml aprotinin, 1 μg/ml pepstatin A, and 1 μg/ml leupeptin), and the sample was bulk incubated with anti-FLAG resin (GenScript, catalog no. L00432) at 4 °C on a turning wheel for 1 h. In general, 0.5 ml bed volume of anti-FLAG resin was sufficient for a sample obtained from 1 L of culture, but the amount of resin was adjusted based on the amount of protein observed on the SDS-PAGE gel. After an hour, the suspension was transferred to a gravity flow column and allowed to drain. The column was washed with 20 CV of TBS300 followed by 20 CV of TBS150. Purified DSIF was eluted in 10 0.5 CV steps with 100 μg/ml 3× FLAG peptide in TBS150. For each elution step, the column was capped and the resin was incubated with the elution buffer for at least 10 min. Fractions were assayed for the presence of DSIF by SDS-PAGE and Coomassie staining. Peak fractions were quantified by running diluted samples on a 3 to 14% Tris-Acetate gradient gel alongside 50, 100, 200, 400, 800, and 1600 ng BSA standards. Gels were Coomassie stained, imaged, and band intensities were quantified using ImageJ. A bovine serum albumin (BSA) standard curve and the Spt5 band intensities were then used to determine the concentration of full-length Spt5 in each fraction. Each quantification gel was run in duplicate and the calculated Spt5 concentrations were verified by running 200 ng of each sample on a separate gel along with a 200 ng BSA standard.

### Expression and purification of NELF

gBlocks (IDT) containing coding sequences of NELF-B, NELF-D, and NELF-E, with a 3x-FLAG tag on the C-terminus of NELF-D and a His_10_ tag on the N-terminus of NELF-E were cloned into a pACEBac1 vector containing the coding sequence for NELF-A using InFusion (Takara catalog no. 638943). The fragments were sequentially cloned into an AvrII restriction site. The pACEBac1_NELF construct was verified by sequencing and transformed into DH10EMBacY cells to generate a NELF bacmid (62).

The recombinant EMBacY bacmid containing the coding sequences for NELF-A, NELF-B, NELF-D-3x-FLAG, and His_10_-NELF-E was transformed into Sf9 cells using FuGENE Transfection reagent (Promega catalog no. E2311). Transfection was confirmed by YFP fluorescence and virus (V_0_) was harvested from transformed cultures in which most cells exhibited YFP fluorescence. Expression of NELF in virus-infected Sf9 cells was confirmed by western blotting against all four subunits.

To purify the NELF complex, 500 ml of Sf9 cell culture (1.5 million cells/ml) were infected with 3 ml of V_1_ NELF baculovirus and incubated at 27 °C. The percentage of infected cells was monitored by tracking YFP fluorescence. After 72 h, the infected cells were harvested by centrifugation at 1000*g* for 10 min and flash frozen for storage at −80 °C. The cells were thawed and suspended in 40 ml of Sf9 lysis buffer (20 mM HEPES, pH 7.6, 500 mM NaCl, 1 % Triton X-100, 10 % glycerol, 5 mM β-mercaptoethanol, 0.1 mM PMSF, 1.6 μg/ml benzamidine-HCl, 1 μg/ml aprotinin, 1 μg/ml pepstatin A, 1 μg/ml leupeptin). The cells were lysed with a Dounce homogenizer (10 strokes of the loose pestle followed by 10 strokes of the tight pestle), and the lysate was cleared by ultracentrifugation at 35,000 rpm in a Ti70 rotor at 4 °C. The cleared lysate was incubated with 1 ml bed volume of Ni-NTA resin (Qiagen catalog no. 30210) on a rotating wheel at 4 °C. After 3.5 h, the resin was collected by centrifugation at 700*g* and washed in bulk with 10 CV of wash buffer (20 mM HEPES, pH 7.6, 500 mM NaCl, 250 mM KCl, 20 mM imidazole, 1 % Triton X-100, 10 % glycerol, 5 mM β-mercaptoethanol, 0.1 mM PMSF, 1.6 μg/ml benzamidine-HCl, 1 μg/ml aprotinin, 1 μg/ml pepstatin A, 1 μg/ml leupeptin). The resin was then transferred to a gravity flow column, drained, and washed with an additional 5 CV of wash buffer. Prior to elution, the column was washed with 5 CV of 0.15 M HEMGK (25 mM HEPES, pH 7.6, 0.1 mM EDTA, 12.5 mM MgCl_2_, 10 % glycerol, 0.15 M KCl). NELF was eluted in 10 0.5 CV steps with 300 mM imidazole in 0.15 M HEMGK.

The fractions eluted from the Ni-NTA column were pooled and mixed with protease inhibitors (final concentrations: 0.1 mM PMSF, 1.6 μg/ml benzamidine-HCl, 1 μg/ml aprotinin, 1 μg/ml pepstatin A, 1 μg/ml leupeptin) before being incubated with 0.75 ml bed volumes of anti-FLAG resin (GenScript, catalog no. L00432) for 2.5 h on a turning wheel at 4 °C. The resin was then transferred to a gravity flow column and washed with ∼60 CV of 0.4 M HEMGK (25 mM HEPES, pH 7.6, 0.1 mM EDTA, 12.5 mM MgCl_2_, 10% glycerol, 0.4 M KCl). NELF was eluted in 10 0.5 CV steps with 100 μg/ml 3× FLAG peptide in 0.15 M HEMGK. For each elution step, the column was capped and the resin was incubated with the elution buffer for at least 10 minutes. Elution fractions were analyzed for the presence of NELF by SDS-PAGE and Coomassie staining.

### Generation of stalled elongation complexes and evaluation of DSIF and NELF binding to Pol II

Stalled elongation complexes were generated using purified *Drosophila* FLAG-Pol II and a fluorescent, tailed DNA template. The DNA template was generated by annealing two synthetic oligonucleotides (Integrated DNA Technologies) containing a 24-nucleotide G-less cassette followed by a 23-nucleotide sequence composed of all four nucleotides. The bottom strand has a Cy5 fluorescent label on the 5′ end and contains an 11-nucleotide overhang that serves as a Pol II initiation site in the presence of an UpG dinucleotide primer (Tri-link or Jena Bioscience). The annealed template was diluted into 50 mM sodium phosphate buffer (pH 7.5) and purified by passage through a hydroxyapatite column equilibrated with the same buffer. The column was washed with 140 mM sodium phosphate buffer (pH 7.5) to remove single-stranded DNA and the double-stranded DNA template was eluted with 250 mM sodium phosphate buffer (pH 7.5). The DNA was ethanol precipitated, dissolved in TE buffer, and desalted using a Bio-6 spin column (Bio-Rad). The sequence of the bottom strand is 5′-Cy5/GCA GGT CGA CTC TAG AGG ATC CCG GGA GTG GAA TGA GAA ATG AAG ATC AAA AAA AAT TA-3′. The sequence of the top strand is 5′- GAT CTT CAT TTC TCA TTC CAC TCC CGG GAT CCT CTA GAG TCG ACC TGC-3′ The final stock concentrations of the DNA template fractions were ∼30 ng/uL to 60 ng/uL.

Elongation complexes were generated as previously described (19, 30, 63) with modifications. A 15 μl premix containing 50 mM HEPES (pH 7.5), 200 mM KCl, 1 mM MnCl_2_, 12% glycerol, 0.5 mM DTT, 0.5 mM UpG, 20 units of RNasin (Promega or VWR), 66 ng/μl BSA, and ∼4 ng of template was incubated with ∼80 ng of *Drosophila* Pol II for 5 min at room temperature. A 5 uL NTP mix containing 0.4 mM ATP, 0.4 mM CTP, 0.4 mM UTP, and 0.02 mM 3′-*O*-methyl-GTP was added to start the reaction. Reactions were incubated at room temperature for 10 min. For each experiment, a reaction containing 10 μg/ml α-amanitin was set up as a negative control. Purified WT or mutant DSIF and NELF diluted in TBS150 were then added to the transcription reactions. BSA (400–600 ng) and 3× FLAG peptide (200 ng) were added to reactions containing no DSIF or NELF. Reactions were incubated at room temperature for 10 min. Torula yeast RNA (5 μg) was then added to each sample to reduce nonspecific binding between NELF and DSIF and the nascent transcript. Reactions were incubated at room temperature for an additional 10 min, then promptly loaded onto a 4% native polyacrylamide gel, which was run at 4 °C as previously described (19, 30). The gel was imaged using Typhoon 9410. NELF recruitment by WT and mutant DSIF was quantified using ImageJ. The percentage of NELF-bound complexes as a fraction of total Pol II-containing complexes was determined and the results for each DSIF mutant were compared to WT DSIF using a two-sample *t* test.

### *In vitro* transcription in nuclear extract

*In vitro* transcription assays to measure the pausing activity of DSIF mutants were performed as previously described (33) with modifications. Nuclear extracts were prepared as described above and immunodepleted with pre-immune serum or anti-Spt5 serum-conjugated protein A Sepharose beads. Extracts underwent three rounds of 2-h depletions at 4 °C. Transcription reactions were performed using a pulse-chase strategy. Briefly, 14 μl of reaction premix (20 mM HEPES pH 7.5, 1 mM DTT, 100 ng *hsp70* promoter-containing DNA template, 2 μg HaeIII-cut *E. coli* DNA, 0.8 units/μl RNasin) were mixed with 16 μl of nuclear extract supplemented with 0.75 μM flavopiridol and 3 to 5 μl of purified DSIF or TBS150. The mixture was incubated at room temperature (∼22 °C) for 20 min to allow pre-initiation complex formation. After 20 min, transcription was initiated by the addition of 3 μl of pulse solution (2.6 mM ATP, 2.6 mM UTP, 1 uL of [α-^32^P] CTP (6000 Ci/mmol, 10 μCi/μl)) to each sample. After 5 min, 2 μl of chase solution (2 mM CTP, 2 mM GTP) was added to each reaction. The chase was allowed to proceed for 5 min and the reaction was stopped with the addition of 200 μl of stop solution (20 mM EDTA, pH 8, 0.2 M NaCl, 1% SDS, 0.25 mg/ml Torula yeast RNA, 0.1 mg/ml proteinase K). Samples were then incubated at 55 °C for 1 h. The samples were then heated to 95 °C for 10 min to inactivate the proteinase K. PMSF was added to each sample to a final concentration of 5 mM to inhibit any residual proteinase K activity. To separate the *hsp70* RNA from irrelevant spuriously labeled tRNA that is present in the nuclear extract, 3 pmol of 3′-biotinylated oligonucleotide complementary to the *hsp70* transcript from +1 to +44 was added to each sample. The samples were then incubated at room temperature (∼22 °C) overnight.

The following day, 150 to 300 μg of washed Dynabeads M-280 Streptavidin (Invitrogen catalog no. 11205D) were added to each sample and incubated for 15 min. The beads were washed once with 300 μl of wash buffer (10 mM NaCl, 10 mM Tris-HCl, pH 7.5, 5 mM EDTA, 0.5 mg/ml yeast tRNA), transferred to a fresh tube, and washed with an additional 300 μl of wash buffer followed by a final wash with 100 μl of wash buffer. Transcripts were eluted in 15 μl of sequencing gel loading buffer (98% deionized formamide, 10 mM EDTA, pH 8.0, 0.025% xylene cyanol FF, 0.025 % bromophenol blue) at 95°C for 5 min and subsequently analyzed on a 10% polyacrylamide gel (19:1 acrylamide:bis-acrylamide) containing 1× TBE (89 mM Tris base, 89 mM Boric acid, 22 mM EDTA disodium salt) and 8 M urea. Gels were pre-run for 30 min in 1× TBE at 50 mA. Five to 7 μl of the sample were loaded per well and gels were run for an additional ∼2.5 h at 50 mA. Gels were transferred to filter paper and dried at 80 °C for 2 h and exposed to a phosphor screen for ∼2 to 5 days. Phosphor screens were imaged using a Typhoon 9410.

### Quantification of the traveling ratio

Nuclear extract transcription gels were quantified using ImageQuant software. For each lane, a traveling ratio was calculated by dividing the total signal from the readthrough bands with the total signal from the paused bands (Fig. 4*E*). Fold increases in traveling ratio were calculated by dividing traveling ratios for mutant DSIF reactions by the traveling ratios for corresponding control WT DSIF reactions. Statistical analysis (1-sample *t* test) was performed using Minitab software.

### Generation of transgenic fly lines

Transgenic fly lines expressing Spt5 mutants were generated using the phiC31 site-specific integration system that has been previously described (30). Mutant Sp5 expression is under the control of a Gal4 upstream activating sequence. To generate these lines, a pUAST-attB plasmid containing an RNAi-resistant Spt5 coding sequence was modified to contain the Spt5 KOW1-Asp, Spt5 KOW4-Asp, Spt5 NGN-S.c., Spt5 NGN-K.p., Spt5 NGN-K.p._R, or Spt5 KOW2-3-S.c. mutant sequences. Mutated sequences were PCR amplified from previously generated pST44-Spt5-Spt4 expression plasmids and cloned into the pUAST-attB-Spt5 plasmid using InFusion (Takara catalog no. 638943). The Spt5 KOW1-Asp, Spt5 NGN-S.c., Spt5 NGN-K.p., Spt5 NGN-K.p._R, and Spt5 KOW2-3-S.c. sequences were amplified using the forward primer 5′-ACATTTACCTGGAGGCCTATAAG-3′ and the reverse primer 5′-GCAGTTGGCTTGCACTCGA-3′ and cloned into a pUAST-attB-Spt5 vector digested with StuI and BstAPI. The Spt5 KOW4-Asp sequence was amplified using the forward primer 5′-ACATTTACCTGGAGGCCTATAAG-3′ and the reverse primer 5′-CGACATAAATCCTAGGCCGC-3′ and cloned into a pUAST-attB-Spt5 vector digested with StuI and AvrII. Plasmids were injected by BestGene Inc into fly line BDSC 24749, which contains an attP site and expresses phiC31 integrase, allowing Spt5 transgene integration into the genome. Flies containing the Spt5 mutant transgenes were homozygosed by inter se matings and selecting red-eyed flies.

The function of each transgenic derivative of Spt5 was tested in two ways. First, they were evaluated to see if they had a dominant negative effect on development in adults when ubiquitously expressed in the presence of the endogenous wild-type Spt5. Flies containing the Spt5 transgene were mated to flies containing an Actin Gal4 transgene on chromosome II over the CyO balancer chromosome (ActGal4/CyO,y+, BDSC 4414). Offspring carrying the CyO balancer have curly wings and do not express the Spt5 transgene due to the absence of Gal4. In contrast, offspring with the homologous chromosome harboring the Actin Gal4 transgene express the transgenic Spt5 throughout development due to the ubiquitous expression of Gal4. The ratio of curly-winged adults to straight-winged adults reveals whether the transgenic Spt5 affects development.

The second test of the function of the transgenic derivatives of Spt5 was to determine the impact of co-expressing the Spt5 derivative along with an Spt5 RNAi that selectively diminishes the endogenous form of Spt5. The Gal4-activated Spt5 transgene was recombined with the Gal4-activated Spt5 RNAi transgene. Mating the resulting flies to ActGal4/CyO,y+ and monitoring the ratio of straight-winged adults to curly-winged adults provided a measure of the transgenic Spt5’s capacity to support development to adulthood.

### Western blotting

Samples were mixed with 4× LDS sample buffer containing 40 mM DTT (NuPAGE catalog no. NP0007), heated to 75°C for 10 min, and run on 3 to 14% Tris-Acetate gradient gels. Proteins were transferred to PVDF membranes, which were probed with rabbit anti-Spt5, anti-NELF-A, anti-NELF-D, anti-NELF-E, guinea pig anti-NELF-B, and mouse anti-Rpb1 (ARNA3, Millipore catalog no. CBL221) primary antibodies to detect Spt5, NELF subunits, and Rpb1 respectively. Blots were probed with anti-rabbit Alexa Fluor 488 (ThermoFisher catalog no. A-11008), anti-guinea pig Alexa Fluor 568 (ThermoFisher catalog no. A-11075), and anti-mouse Alexa Fluor 647 (ThermoFisher catalog no. A32728) secondary antibodies. Blots were imaged using a Typhoon 9410.

To evaluate the expression of UAS-Spt5 in transgenic flies, heads from five frozen flies were transferred to a microfuge and ground with a pipette tip. The contents of the tube were then suspended in 50 uL of 1× LDS sample buffer (NuPAGE catalog no. NP0007), 1.25 uL protease inhibitor cocktail (1.6 μg/ml benzamidine-HCl, 1 μg/ml aprotinin, 1 μg/ml pepstatin A, and 1 μg/ml leupeptin), and 6.25 uL 1M DTT. Samples were heated at 95 °C for 10 min and centrifuged at 16,000*g* for an additional 10 min. The supernatant was transferred to a fresh tube and used for western blotting. Samples were run on a 3 to 14% Tris-Acetate gradient gel. Proteins were transferred to a nitrocellulose membrane which was then probed with mouse anti-FLAG M2 primary antibody (Sigma-Aldrich catalog no. F1804–50UG) and anti-mouse Alexa Fluor 488 secondary antibody (ThermoFisher catalog no. A32723) or rabbit anti-Spt5 primary antibody and anti-rabbit Alexa Fluor 647 secondary antibody (ThermoFisher catalog no. A32733) to detect the FLAG-tagged ectopic Spt5 and the endogenous Spt5 respectively. Blots were also probed with rabbit anti-M1BP primary and anti-rabbit Alexa Fluor 647 (ThermoFisher catalog no. A27040) antibody as a loading control.

### Additional software used

Structural images and structural alignments were generated using PyMol. Amino acid sequence alignments were performed using SeaView alignment software (64). Structural models of Spt4 and the Spt5 NGN domains of *C. elegans* and *Drosophila melanogaster* were generated using AlphaFold (65, 66). Graphs shown in this manuscript were generated using Microsoft Excel and GraphPad Prism 9.

## Data availability

All data are contained within the manuscript.

## Supporting information

This article contains supporting information (30).

## Conflict of interest

The authors declare that they have no conflicts of interest with the contents of this article.
