# Supplementary material for: Assessment of the roles of Spt5-nucleic acid contacts in promoter proximal pausing of RNA polymerase II
Source: J Biol Chem. 2023 Jul 28;299(9):105106. doi: 10.1016/j.jbc.2023.105106 (PMC10482750; doi:10.1016/j.jbc.2023.105106)
Supplement: Supporting information [file mmc1.pdf]

Assessment of the roles of Spt5-nucleic acid contacts in promoter proximal pausing of RNA polymerase II

Roberta Dollinger[1], Eilene B. Deng[1], Josie Schultz[1], Sharon Wu[1], Haley R. Deorio[1,2], and David S. Gilmour[1,3]

[1]Center for Eukaryotic Gene Regulation, Department of Biochemistry and Molecular Biology, Pennsylvania State University, University Park, Pennsylvania, USA

[2]Department of Biomolecular Chemistry, University of Wisconsin-Madison, Madison, Wisconsin, USA

[3]Corresponding author: dsg11@psu.edu

Supporting information: The supporting information below contains additional data and replicates of experiments described in the main text.

Figure S1: Western blot verification of FLAG-Rpb1 Pol II and NELF
Figure S2: DSIF mutants bind Pol II
Figure S3: DSIF mutants recruit NELF to the elongation complex
Figure S4: NELF recruitment by DSIF mutants
Figure S5: DSIF mutants have impaired pausing activity in nuclear extract
Figure S6: Pausing activity of Spt5 NGN mutants
Figure S7: The KOW2-3 domain is involved in NELF recruitment to the elongation complex
Figure S8: Expression of UAS-Spt5 mutants in *Drosophila* heads

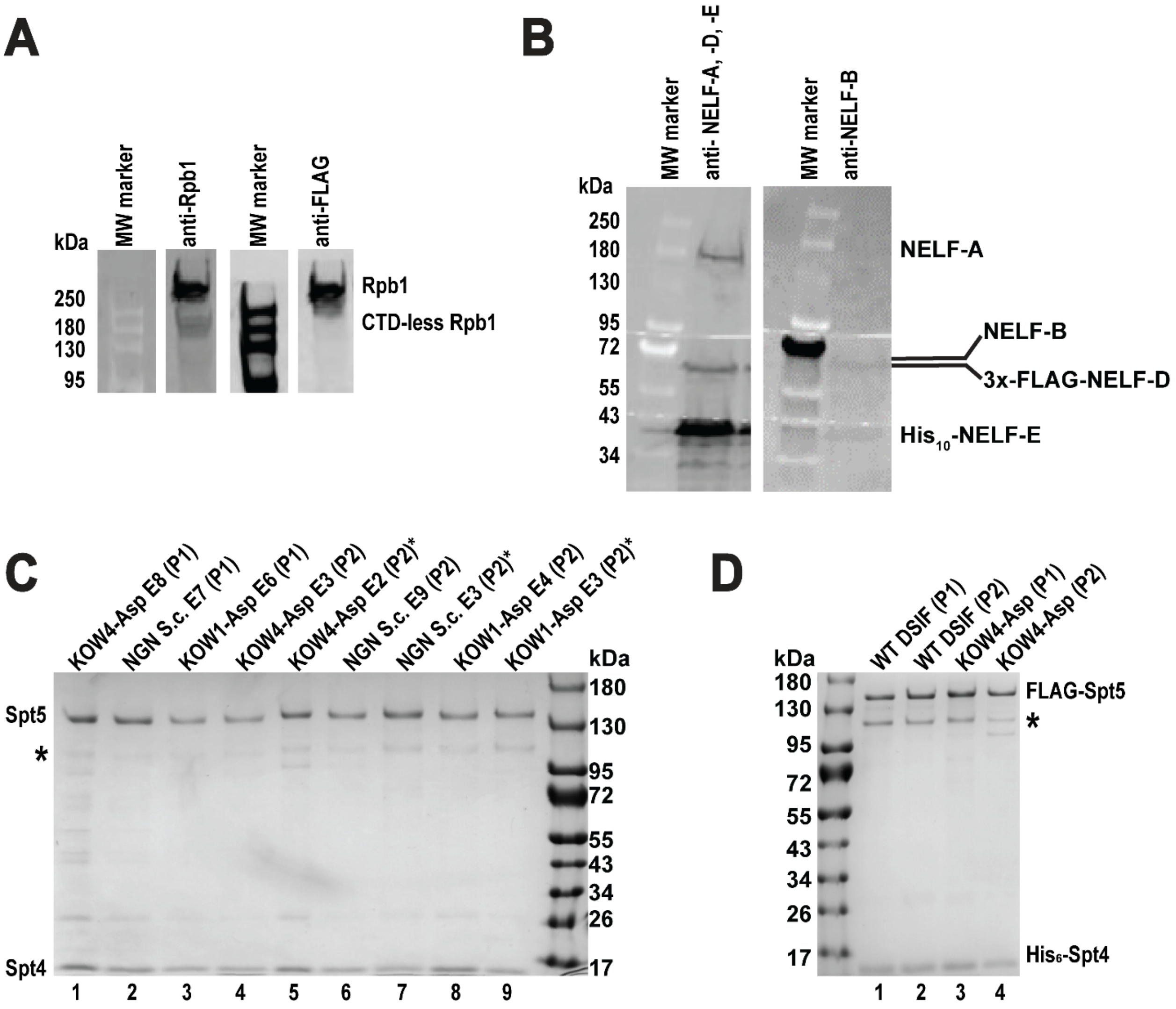

Figure S1: Western blot verification of FLAG-Rpb1 Pol II & NELF and Coomassie evaluation of DSIF mutants A) Western blot of FLAG-Rpb1 Pol II eluted from a POROS Heparin column loaded with *Drosophila* nuclear extract. A portion of the Rpb1 lacks a C-terminal domain. Further purificaiton with anti-FLAG column removes this degraded Rpb1. B) Western blot against the four subunits of NELF in lysate from NELF baculovirus-infected Sf9 cells. C) Coomassie stained 3-14% Tris-Acetate gel showing different preparations of KOW4-Asp, NGN S.c., and KOW1-Asp mutants. 200 ng of Spt5 were loaded in each lane. Fractions marked with an asterisk were used in EMSAs shown in Figures S2A (bottom) and S2E (bottom). A ~100 kDa Spt5 degradation product lacking the acidic N-terminal region appears in each preparation (asterisk). D) Coomassie stained 3-14% Tris-Acetate gel showing different preparations of WT DSIF and KOW4-Asp. 200 ng of Spt5 were loaded in each lane. Fractions were used in EMSAs shown in Figure S2B (bottom). A ~100 kDa Spt5 degradation product lacking the acidic N-terminal region appears in each preparation (asterisk).

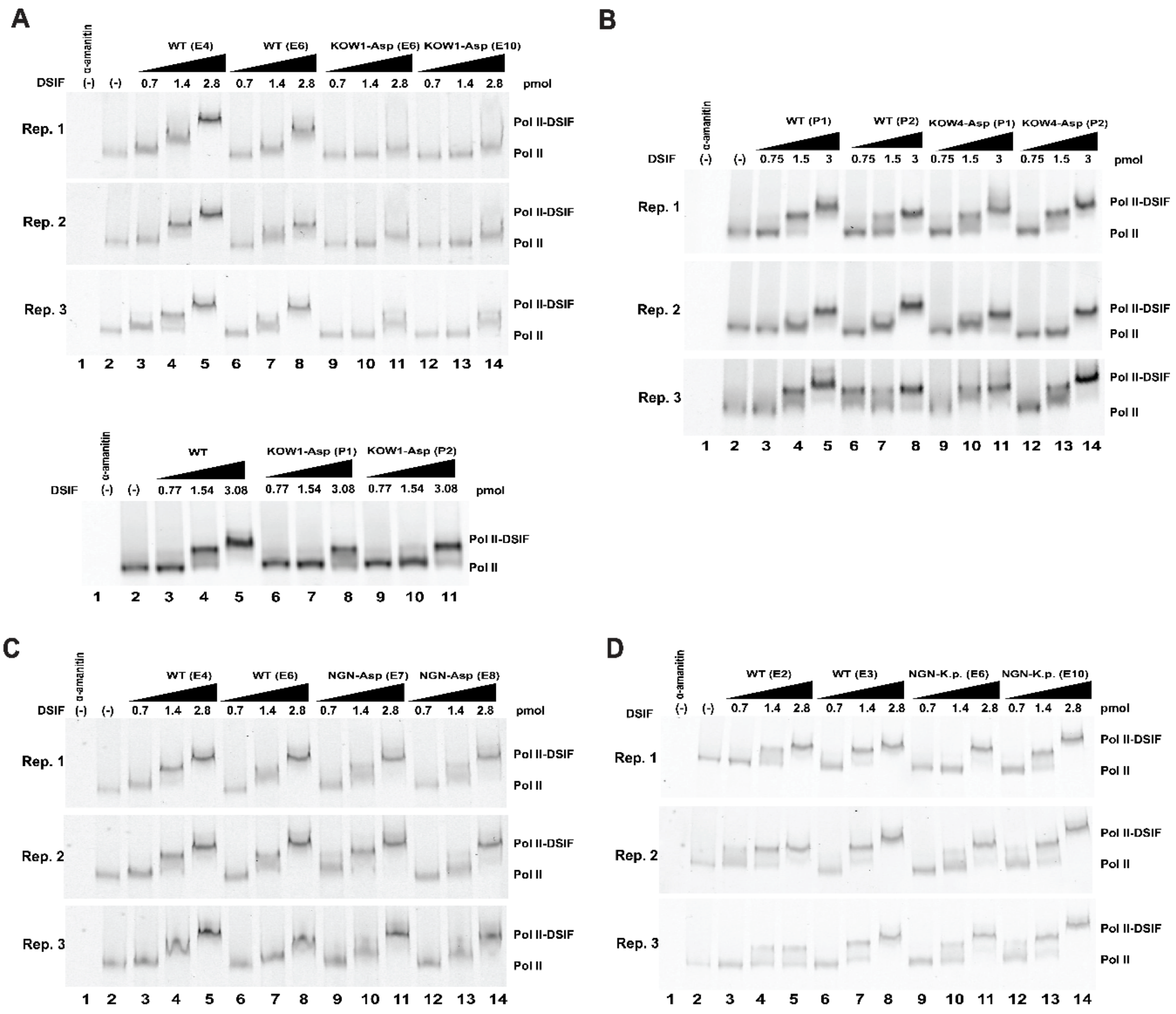
A
α-amanitin
WT (E4)
WT (E6)
KOW1-Asp (E6)
KOW1-Asp (E10)
DSIF (-) (-) 0.7 1.4 2.8 0.7 1.4 2.8 0.7 1.4 2.8 0.7 1.4 2.8 pmol
Rep. 1
Rep. 2
Rep. 3
Pol II-DSIF
Pol II
1 2 3 4 5 6 7 8 9 10 11 12 13 14
α-amanitin
WT
KOW1-Asp (P1)
KOW1-Asp (P2)
DSIF (-) (-) 0.77 1.54 3.08 0.77 1.54 3.08 0.77 1.54 3.08 pmol
Pol II-DSIF
Pol II
1 2 3 4 5 6 7 8 9 10 11
B
α-amanitin
WT (P1)
WT (P2)
KOW4-Asp (P1)
KOW4-Asp (P2)
DSIF (-) (-) 0.75 1.5 3 0.75 1.5 3 0.75 1.5 3 0.75 1.5 3 pmol
Rep. 1
Rep. 2
Rep. 3
Pol II-DSIF
Pol II
1 2 3 4 5 6 7 8 9 10 11 12 13 14
C
α-amanitin
WT (E4)
WT (E6)
NGN-Asp (E7)
NGN-Asp (E8)
DSIF (-) (-) 0.7 1.4 2.8 0.7 1.4 2.8 0.7 1.4 2.8 0.7 1.4 2.8 pmol
Rep. 1
Rep. 2
Rep. 3
Pol II-DSIF
Pol II
1 2 3 4 5 6 7 8 9 10 11 12 13 14
D
α-amanitin
WT (E2)
WT (E3)
NGN-K.p. (E6)
NGN-K.p. (E10)
DSIF (-) (-) 0.7 1.4 2.8 0.7 1.4 2.8 0.7 1.4 2.8 0.7 1.4 2.8 pmol
Rep. 1
Rep. 2
Rep. 3
Pol II-DSIF
Pol II
1 2 3 4 5 6 7 8 9 10 11 12 13 14

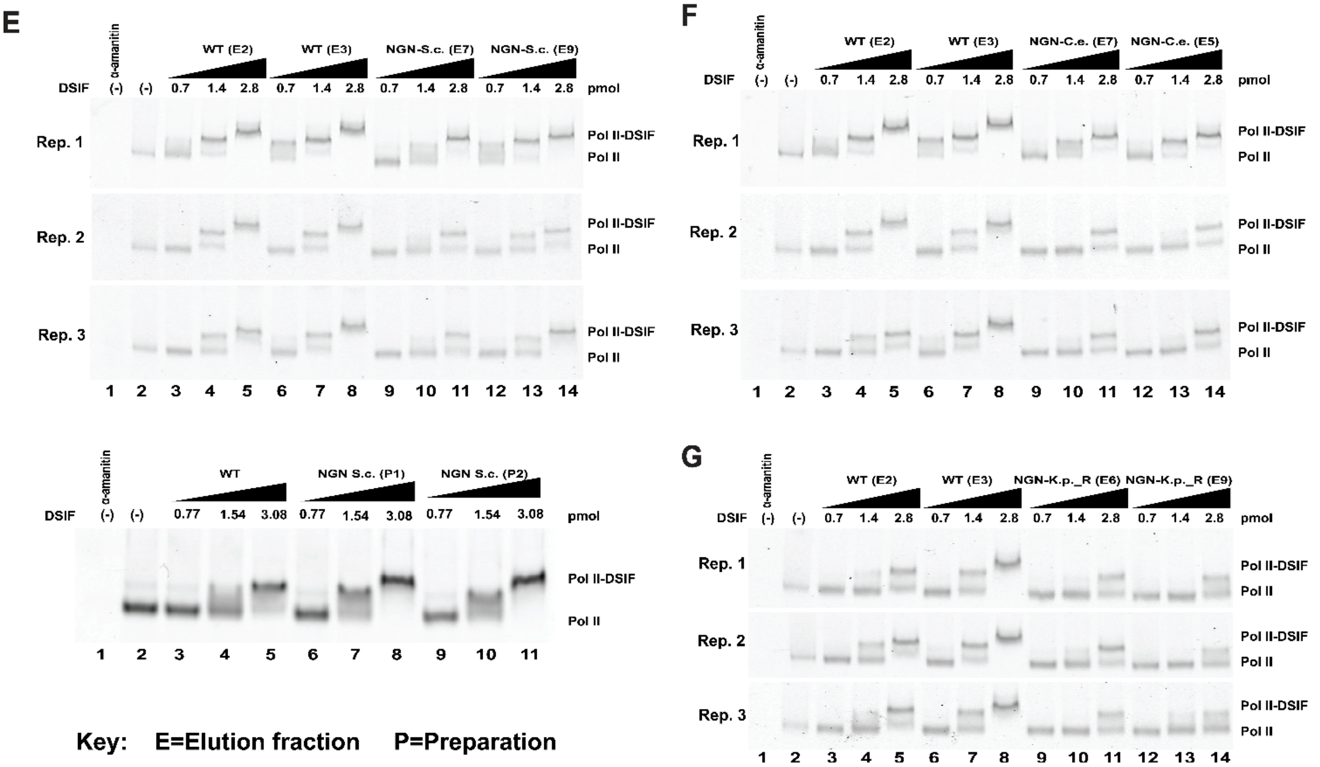

Figure S2: DSIF mutants bind Pol II Stalled elongation complexes were generated as in Fig. 3. For each mutant, two separate WT DSIF fractions were compared to two separate mutant fractions. "Rep" indicates experimental replicates. All replicates are shown here to facilitate comparison. Representative replicates are also presented in the main figures. A) KOW1-Asp Pol II binding. Top gels show comparison between two different fractions of the same preparation (E6 and E10). The gel for Rep. 1 can also be found in Fig. 3B. Bottom gel shows comparison between two different KOW1-Asp preparations (P1 and P2). B) KOW4-Asp Pol II binding. Comparison of two separate preparations of WT DSIF and KOW4-Asp (P1 and P2). Rep. 1 gel can also be found in Fig. 3D. The KOW4-Asp P1 fraction was used for subsequent experiments to test NELF binding (Fig. S3E) and pausing in nuclear extract (Fig. S5F). C) NGN-Asp Pol II binding. Two fractions were tested (E7 and E8). Rep. 1 gel can also be found in Fig. 3C. D) NGN-K.p. Pol II binding. Two fractions were tested (E6 and E10). Rep. 1 can also be found in Fig. 5B. E) NGN-S.c. Pol II binding. Top gels show comparison between two different fractions of the same preparation (E7 and E9). Rep. 1 can also be found in Fig. 5C. Bottom panel shows comparison between two different NGN-S.c. preparations (P1 and P2). F) NGN-C.e. Pol II binding. Two fractions were tested (E7 and E5). E5 was used for subsequent NELF binding and pausing experiments. G) NGN-K.p._R Pol II binding. Two fractions were tested (E6 and E9). The apparent difference in Pol II binding between WT E3 and the NGN-K.p._R fractions can be attributed to variation in Spt5 quantification (see Fig. 2, cf lanes 8, 13-14). WT E2 and NGN-K.p. E6 were selected for subsequent analysis in nuclear extract.

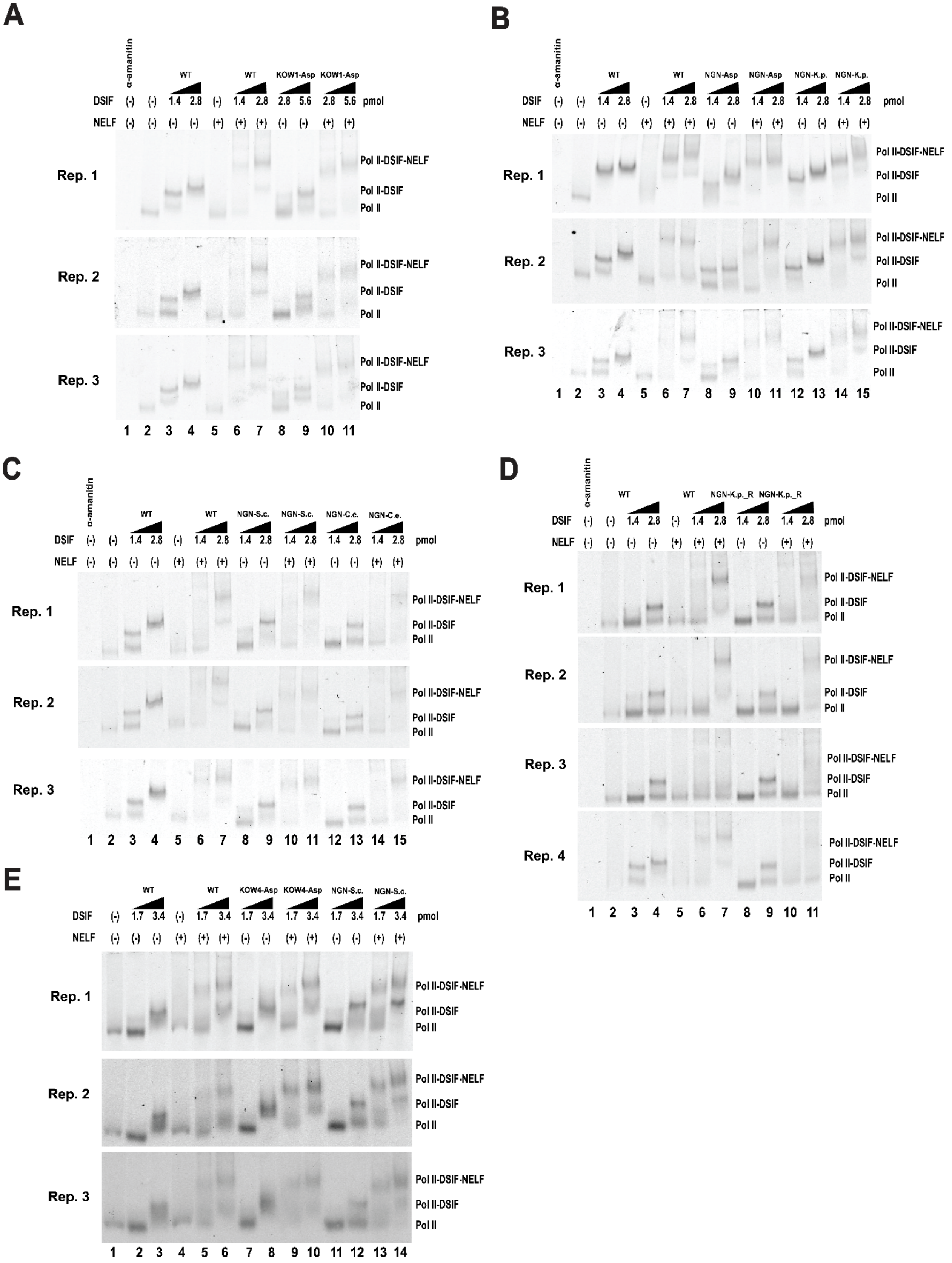
A
α-amanitin
WT
WT
KOW1-Asp
KOW1-Asp
DSIF (-) (-) 1.4 2.8 (-) 1.4 2.8 2.8 5.6 2.8 5.6 pmol
NELF (-) (-) (-) (-) (+) (+) (+) (-) (-) (+) (+)
Rep. 1
Rep. 2
Rep. 3
Pol II-DSIF-NELF
Pol II-DSIF
Pol II
1 2 3 4 5 6 7 8 9 10 11
B
α-amanitin
WT
WT
NGN-Asp
NGN-Asp
NGN-K.p.
NGN-K.p.
DSIF (-) (-) 1.4 2.8 (-) 1.4 2.8 1.4 2.8 1.4 2.8 1.4 2.8 1.4 2.8 pmol
NELF (-) (-) (-) (-) (+) (+) (+) (-) (-) (+) (+) (-) (-) (+) (+)
Rep. 1
Rep. 2
Rep. 3
Pol II-DSIF-NELF
Pol II-DSIF
Pol II
1 2 3 4 5 6 7 8 9 10 11 12 13 14 15
C
α-amanitin
WT
WT
NGN-S.c.
NGN-S.c.
NGN-C.e.
NGN-C.e.
DSIF (-) (-) 1.4 2.8 (-) 1.4 2.8 1.4 2.8 1.4 2.8 1.4 2.8 1.4 2.8 pmol
NELF (-) (-) (-) (-) (+) (+) (+) (-) (-) (+) (+) (-) (-) (+) (+)
Rep. 1
Rep. 2
Rep. 3
Pol II-DSIF-NELF
Pol II-DSIF
Pol II
1 2 3 4 5 6 7 8 9 10 11 12 13 14 15
D
α-amanitin
WT
WT
NGN-K.p._R
NGN-K.p._R
DSIF (-) (-) 1.4 2.8 (-) 1.4 2.8 1.4 2.8 1.4 2.8 pmol
NELF (-) (-) (-) (-) (+) (+) (+) (-) (-) (+) (+)
Rep. 1
Rep. 2
Rep. 3
Rep. 4
Pol II-DSIF-NELF
Pol II-DSIF
Pol II
1 2 3 4 5 6 7 8 9 10 11
E
WT
WT
KOW4-Asp
KOW4-Asp
NGN-S.c.
NGN-S.c.
DSIF (-) 1.7 3.4 (-) 1.7 3.4 1.7 3.4 1.7 3.4 1.7 3.4 1.7 3.4 pmol
NELF (-) (-) (-) (+) (+) (+) (-) (-) (+) (+) (-) (-) (+) (+)
Rep. 1
Rep. 2
Rep. 3
Pol II-DSIF-NELF
Pol II-DSIF
Pol II
1 2 3 4 5 6 7 8 9 10 11 12 13 14

Figure S3: DSIF mutants recruit NELF to the elongation complex Stalled elongation complexes were generated as in Fig. 3. “Rep” indicates experimental replicates. All replicates are shown here to facilitate comparison. Representative replicates are also shown in the main figures. A) Pol II and NELF binding of KOW1-Asp. Rep. 1 can also be found in Fig. 3E. B) Pol II and NELF binding of NGN-Asp and NGN-K.p. Rep. 1 can also be found in Figs. 3G (NGN-Asp only) and 5D. C) Pol II and NELF binding of NGN-S.c. (fraction E9) and NGN-C.e. compared to WT DSIF (fraction E3). Rep. 1 can also be found in Fig. 5E (NGN-S.c. only). D) Pol II and NELF binding of NGN-K.p._R. Rep. 2 can also be found in Fig. 5F. E) NELF binding for KOW4-Asp and NGN-S.c. (fraction E9) mutants compared to WT DSIF (fraction P2). Rep. 1 can also be found in Fig. 5E. Experiments in panels A-C and E were performed in triplicate. The experiment in Panel D was performed with four replicates. Quantification of NELF binding can be found in Fig. S4.

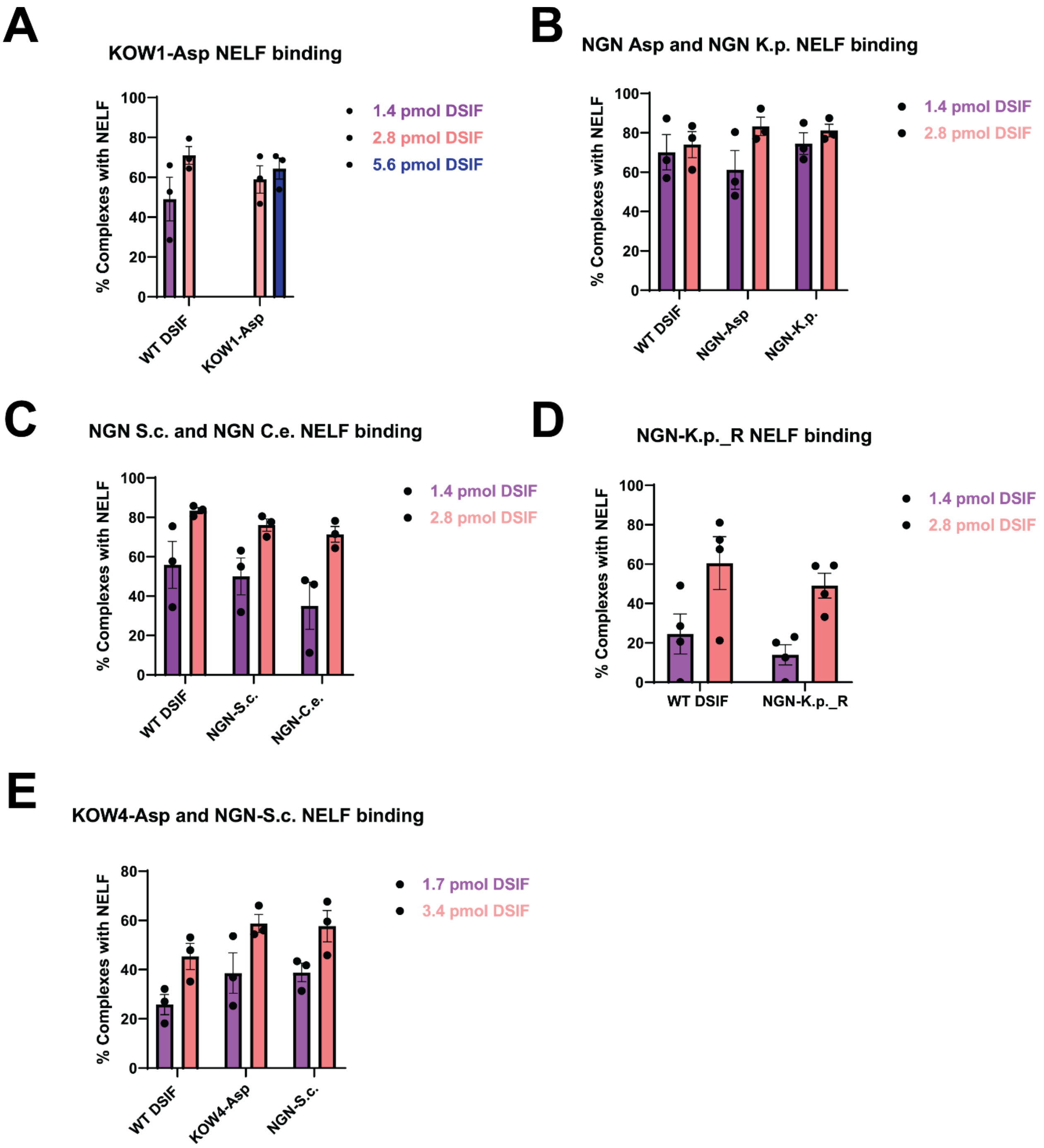

Figure S4: NELF recruitment by DSIF mutants Quantification of gels shown in Fig. S3. Bands were quantified using ImageJ and the percentage of Pol II-DSIF-NELF complex as a fraction of total complexes containing Pol II was calculated for each lane. WT and mutant values were compared by two-sample t-test. P>0.1 for all mutants at all DSIF concentrations. A) Pol II and NELF binding of KOW1-Asp. Note that 2.8 pmol and 5.6 pmol of KOW1-Asp were tested because binding of the KOW1-Asp mutant to the elongation complex was weaker than that of WT DSIF. B) Pol II and NELF binding of NGN-Asp and NGN-K.p. C) Pol II and NELF binding of NGN-S.c. and NGN-C.e. D) Pol II and NELF binding of NGN-K.p._R. E) NELF binding for KOW4-Asp NGN-

S.c. (fraction E9) mutants. Experiments in panels A-C and E were performed in triplicate. The experiment in Panel D was performed with four replicates.

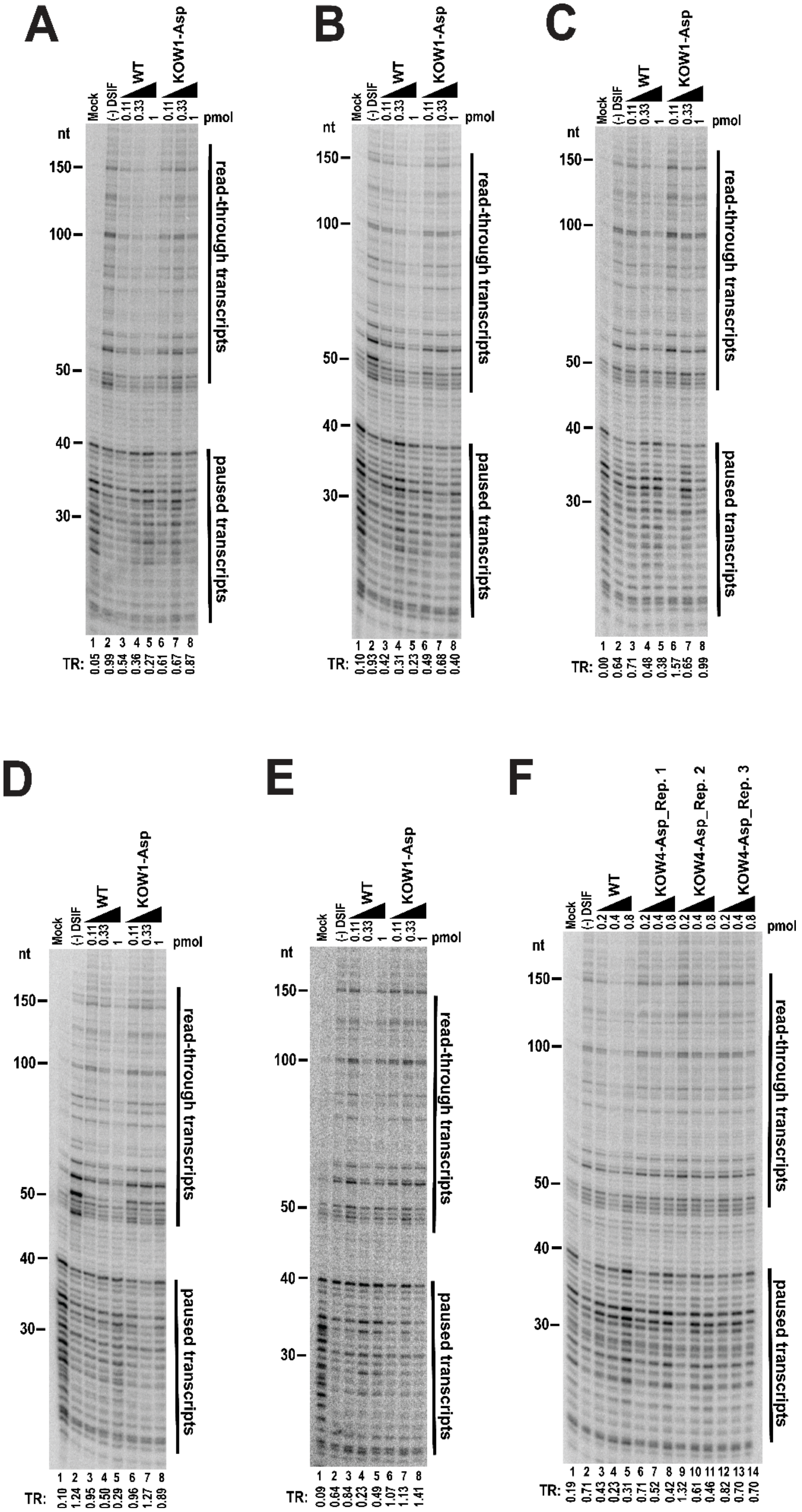
A
B
C
D
E
F
Mock
(-) DSIF
WT
KOW1-Asp
KOW4-Asp_Rep. 1
KOW4-Asp_Rep. 2
KOW4-Asp_Rep. 3
pmol
nt
150
100
50
40
30
read-through transcripts
paused transcripts
TR:
0.05 0.99 0.54 0.36 0.27 0.61 0.67 0.87
0.10 0.93 0.42 0.31 0.23 0.49 0.68 0.40
0.00 0.64 0.71 0.48 0.38 1.57 1.65 0.99
0.10 1.24 0.95 0.50 0.29 0.96 1.27 0.89
0.09 0.64 0.84 0.23 0.49 1.07 1.13 1.41
0.19 0.71 0.43 0.23 0.31 0.71 0.52 0.42 1.32 0.61 0.46 0.82 0.70 0.70

Figure S5: DSIF mutants have impaired pausing activity in nuclear extract Transcription reactions were set up as in Fig.4 using DSIF-depleted *Drosophila* nuclear extract with mock depleted extract as a control. Traveling ratios are listed under each lane. A-E) Pausing activity of KOW1-Asp. F) Pausing activity of KOW4-Asp. The image in panel A can also be found in Fig. 4C (without raw traveling ratios listed) and the image in panel F can also be found in Fig. 4D (without raw traveling ratios listed).

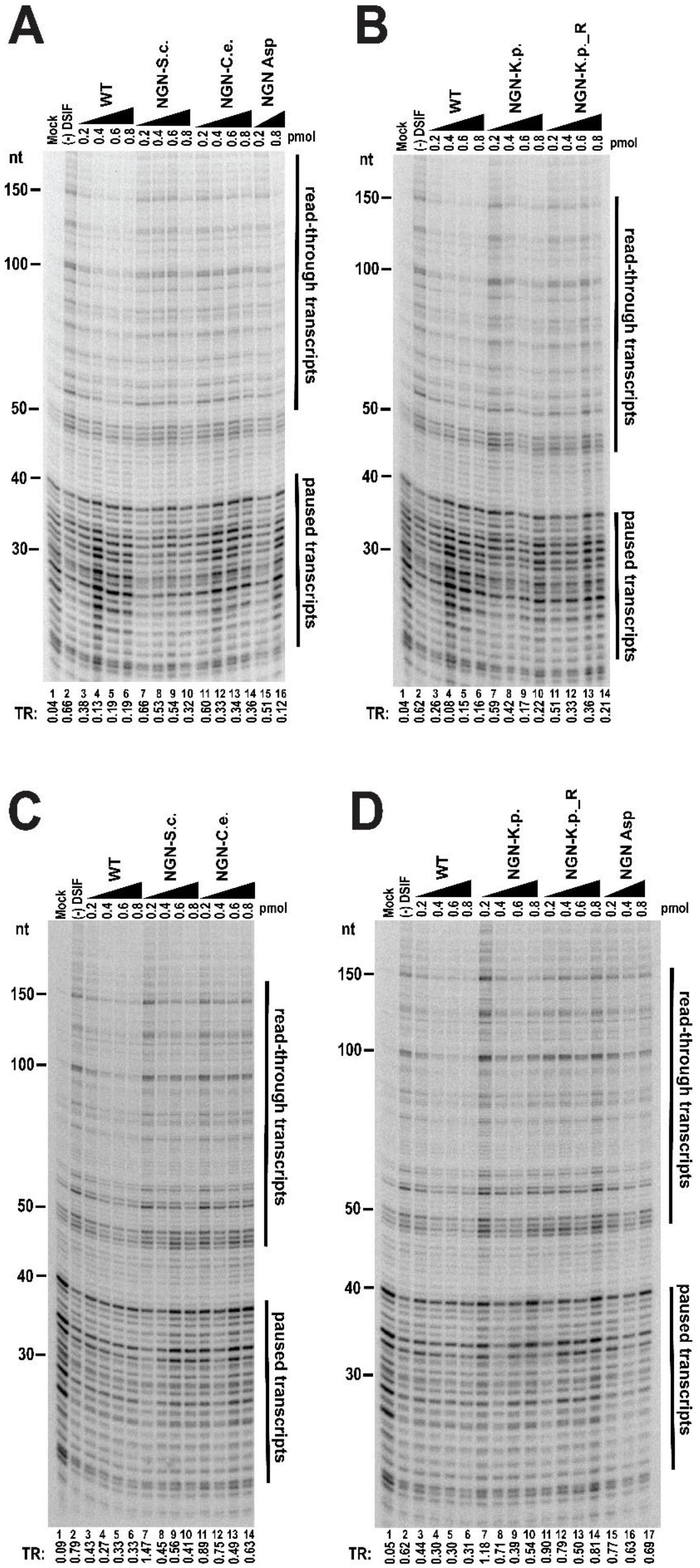
A
B
C
D
Mock
(-) DSIF
WT
NGN-S.c.
NGN-C.e.
NGN Asp
NGN-K.p.
NGN-K.p._R
pmol
nt
read-through transcripts
paused transcripts
TR:

E

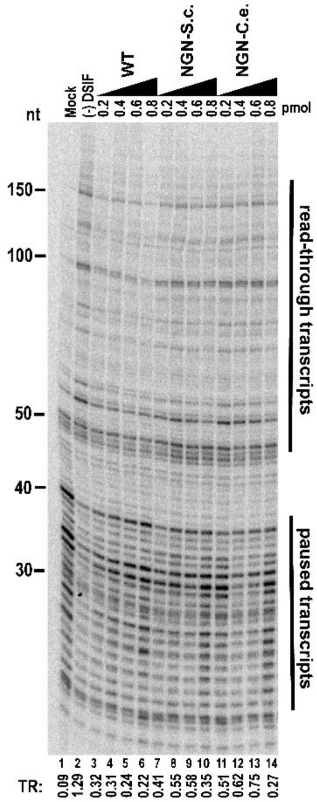
nt
Mock
(-) DSIF
WT
NGN-S.c.
NGN-C.e.
pmol
150
100
50
40
30
read-through transcripts
paused transcripts
TR:

F

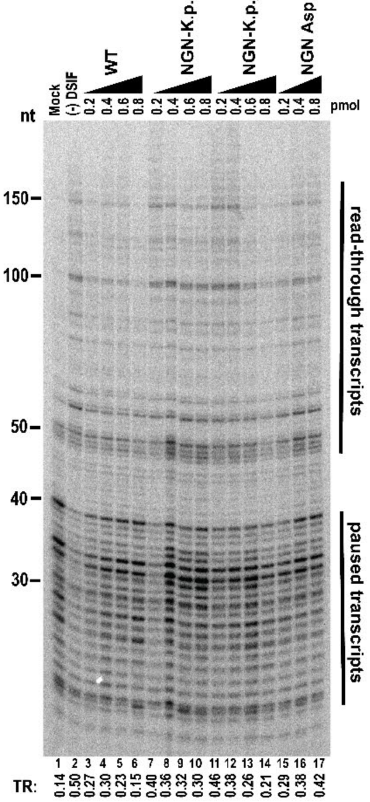
nt
Mock
(-) DSIF
WT
NGN-K.p.
NGN-K.p._R
NGN Asp
pmol
150
100
50
40
30
read-through transcripts
paused transcripts
TR:

G

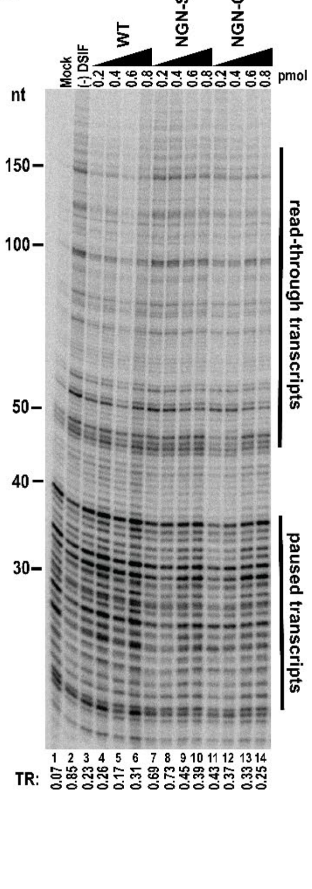
nt
Mock
(-) DSIF
WT
NGN-S.c.
NGN-C.e.
pmol
150
100
50
40
30
read-through transcripts
paused transcripts
TR:

H

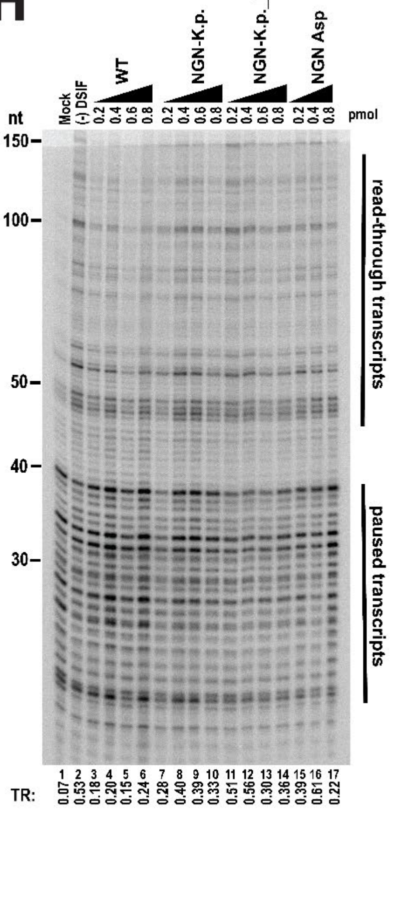
nt
Mock
(-) DSIF
WT
NGN-K.p.
NGN-K.p._R
NGN Asp
pmol
150
100
50
40
30
read-through transcripts
paused transcripts
TR:

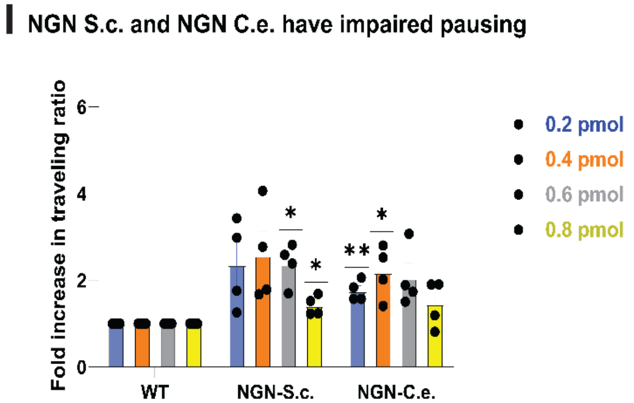

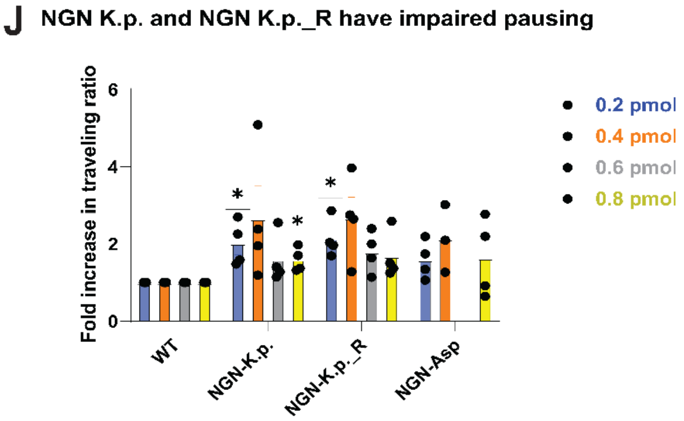

Figure S6: Pausing activity of Spt5 NGN mutants Transcription reactions were set up as in Fig.4 using DSIF-depleted *Drosophila* nuclear extract with mock depleted extract as a control. Traveling ratios are listed under each lane. A) Pausing activity of NGN-S.c., NGN-C.e., and NGN-Asp. B) Pausing activity of NGN-K.p. and NGN-K.p._R. C) Pausing activity of NGN-S.c. and NGN-C.e. D) Pausing activity of NGN-K.p., NGN-K.p._R, and NGN-Asp. E) Pausing activity of NGN-S.c. and NGN-C.e. F) Pausing activity of NGN-K.p., NGN-K.p._R, and NGN-Asp. F) Pausing activity of NGN-S.c. and NGN-C.e. H) Pausing activity of NGN-K.p., NGN-K.p._R, and NGN-Asp. I) Fold increase in traveling ratio for NGN-S.c and NGN-C.e. (panels A, C, E, G). One star indicates $p<0.05$. Error bars indicate standard error of the mean. J) Fold increase in traveling ratio for NGN-K.p., NGN-K.p._R, and NGN-Asp (panels A, D, F, H). One asterisk indicates $p<0.05$ (n=4, n=3 for 0.4 pmol NGN-Asp). Error bars indicate standard error of the mean. The image in panel A can also be found in Fig. 6A (NGN-S.c. lanes only, raw traveling ratios not listed) and the image in panel B can also be found in Fig. 6B (traveling ratios not listed).

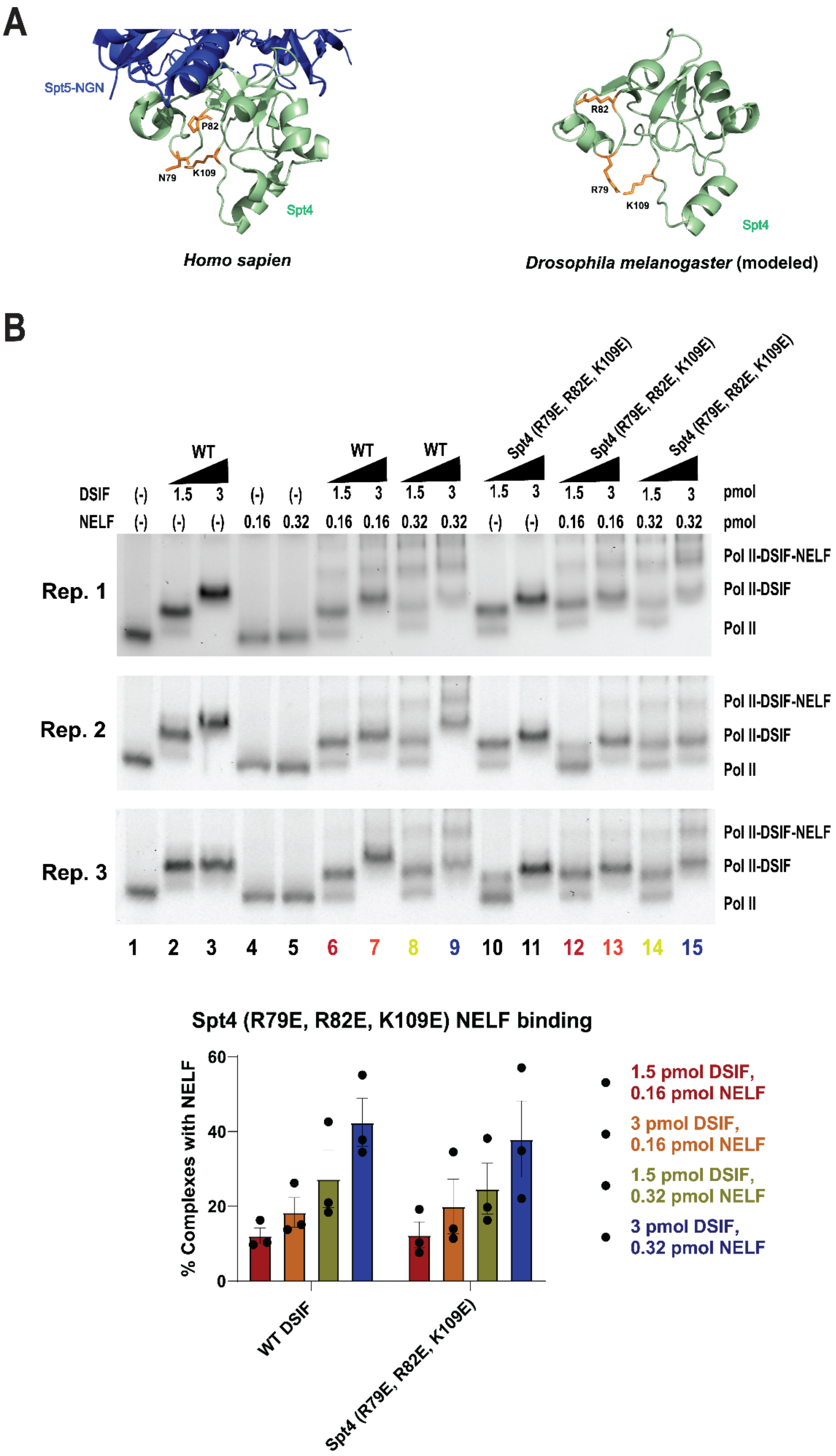
A
Spt5-NGN
P82
N79
K109
Spt4
Homo sapien
R82
R79
K109
Spt4
Drosophila melanogaster (modeled)
B
WT
WT
WT
Spt4 (R79E, R82E, K109E)
Spt4 (R79E, R82E, K109E)
Spt4 (R79E, R82E, K109E)
DSIF (-) 1.5 3 (-) (-) 1.5 3 1.5 3 1.5 3 1.5 3 1.5 3 pmol
NELF (-) (-) (-) 0.16 0.32 0.16 0.16 0.32 0.32 (-) (-) 0.16 0.16 0.32 0.32 pmol
Rep. 1
Rep. 2
Rep. 3
Pol II-DSIF-NELF
Pol II-DSIF
Pol II
1 2 3 4 5 6 7 8 9 10 11 12 13 14 15
Spt4 (R79E, R82E, K109E) NELF binding
% Complexes with NELF
60
40
20
0
WT DSIF
Spt4 (R79E, R82E, K109E)
1.5 pmol DSIF, 0.16 pmol NELF
3 pmol DSIF, 0.16 pmol NELF
1.5 pmol DSIF, 0.32 pmol NELF
3 pmol DSIF, 0.32 pmol NELF

Figure S7: The KOW2-3 domain is involved in NELF recruitment to the elongation complex A) In the human paused elongation structure, the NELF-A crosslinking Spt4 K109 residue is found in the same spatial pocket as P62 and N79, which are positively charged arginine residues in *Drosophila.* PDB ID: 6GML. *B*) Stalled elongation complexes were generated as in Fig. 3.) Pol II binding and NELF recruitment for Spt4 (R79E, R82E, K109E) (top) and quantification of NELF recruitment (bottom). Two-sample t-test comparison of WT DSIF and Spt4 (R79E, R82e, K109E) showed no difference between the WT and mutant ($p>0.7$).

A

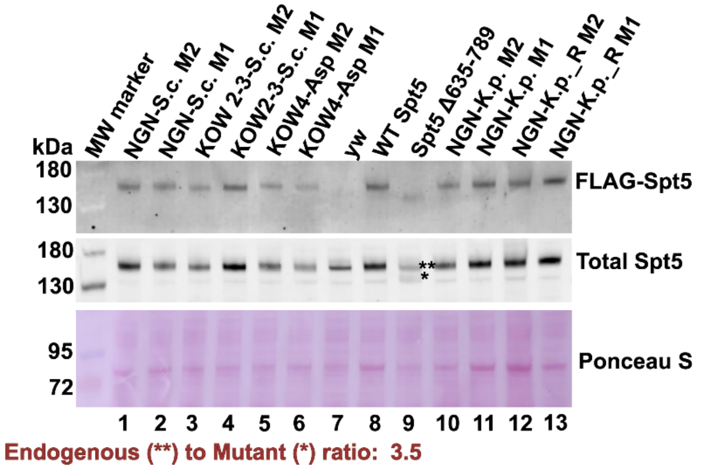

MW marker
NGN-S.c. M2
NGN-S.c. M1
KOW 2-3-S.c. M2
KOW2-3-S.c. M1
KOW4-Asp M2
KOW4-Asp M1
yw
WT Spt5
Spt5 Δ635-789
NGN-K.p. M2
NGN-K.p. M1
NGN-K.p._R M2
NGN-K.p._R M1
kDa
180
130
FLAG-Spt5
180
130
Total Spt5
**
*
95
72
Ponceau S
1 2 3 4 5 6 7 8 9 10 11 12 13
Endogenous (**) to Mutant (*) ratio: 3.5

B

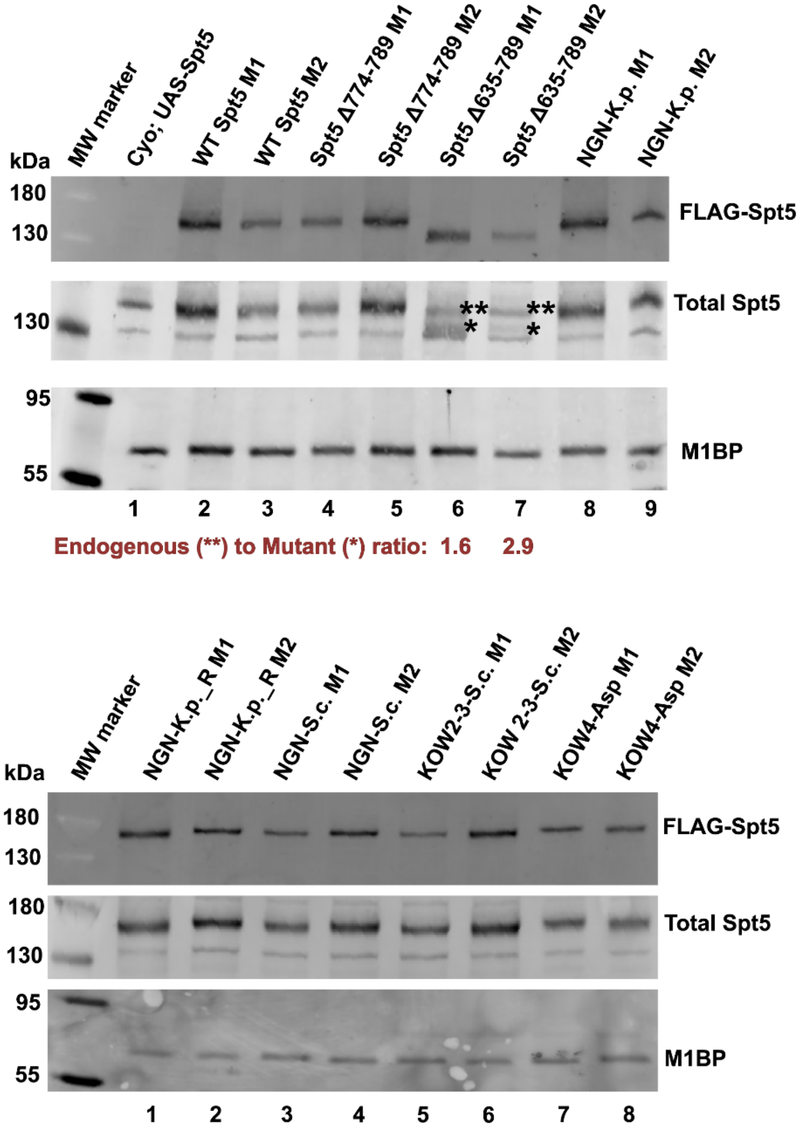

MW marker
Cyo; UAS-Spt5
WT Spt5 M1
WT Spt5 M2
Spt5 Δ774-789 M1
Spt5 Δ774-789 M2
Spt5 Δ635-789 M1
Spt5 Δ635-789 M2
NGN-K.p. M1
NGN-K.p. M2
kDa
180
130
FLAG-Spt5
130
Total Spt5
**
*
**
*
95
55
M1BP
1 2 3 4 5 6 7 8 9
Endogenous (**) to Mutant (*) ratio: 1.6 2.9
MW marker
NGN-K.p._R M1
NGN-K.p._R M2
NGN-S.c. M1
NGN-S.c. M2
KOW2-3-S.c. M1
KOW 2-3-S.c. M2
KOW4-Asp M1
KOW4-Asp M2
kDa
180
130
FLAG-Spt5
180
130
Total Spt5
95
55
M1BP
1 2 3 4 5 6 7 8

Figure S8: Expression of UAS-Spt5 mutants in *Drosophila* heads UAS-Spt5 mutant flies were mated to Act-Gal4/CyO flies to generage Act-Gal4; UAS-Spt5 (straight wing) and CyO; UAS-Spt5 (curly wing) flies. Heads from straight-winged progeny were used for western blotting. M1 and M2 represent two independent lines. Straight winged flies express both the endogenous WT Spt5 as well as the ectopic mutant Spt5; the mutant Spt5 is contains a FLAG tag, allowing for detection by a mouse anti-FLAG antibody. The Spt5 Δ635-789 mutant was used to approximate the ratio of endogenous (two asterisks) to ectopic mutant (one asterisk) Spt5 (shown in red). A) Heads from yw flies were used as a negative control. The Spt5 Δ635-789 mutant is a mutant with an internal deletion that our group has previously epressed in flies[30].The endogenous Spt5 was detected using a rabbit anti-Spt5 antibody. The blot was stained with Ponceau S as a loading control. B) Heads from curly-winged flies were used as a negative control. The Spt5 Δ635-789 and Spt5 Δ774-789 mutants are mutants with an internal deletion that our group has previously epressed in flies[30]. The transcription factor M1BP was detected as a loading control.
